# Tuning the Mammalian Circadian Clock: Robust Synergy of Two Loops

**DOI:** 10.1371/journal.pcbi.1002309

**Published:** 2011-12-15

**Authors:** Angela Relógio, Pal O. Westermark, Thomas Wallach, Katja Schellenberg, Achim Kramer, Hanspeter Herzel

**Affiliations:** 1 Institute for Theoretical Biology, Humboldt University, Berlin, Germany; 2 Laboratory of Chronobiology, Institute of Medical Immunology Charité - Universitätsmedizin Berlin, Berlin, Germany; NNF Center for Protein Research, Denmark

## Abstract

The circadian clock is accountable for the regulation of internal rhythms in most living organisms. It allows the anticipation of environmental changes during the day and a better adaptation of physiological processes. In mammals the main clock is located in the suprachiasmatic nucleus (SCN) and synchronizes secondary clocks throughout the body. Its molecular constituents form an intracellular network which dictates circadian time and regulates clock-controlled genes. These clock-controlled genes are involved in crucial biological processes including metabolism and cell cycle regulation. Its malfunction can lead to disruption of biological rhythms and cause severe damage to the organism. The detailed mechanisms that govern the circadian system are not yet completely understood. Mathematical models can be of great help to exploit the mechanism of the circadian circuitry. We built a mathematical model for the core clock system using available data on phases and amplitudes of clock components obtained from an extensive literature search. This model was used to answer complex questions for example: how does the degradation rate of *Per* affect the period of the system and what is the role of the ROR/*Bmal*/REV-ERB (RBR) loop? Our findings indicate that an increase in the RNA degradation rate of the clock gene *Period* (*Per*) can contribute to increase or decrease of the period - a consequence of a non-monotonic effect of *Per* transcript stability on the circadian period identified by our model. Furthermore, we provide theoretical evidence for a potential role of the RBR loop as an independent oscillator. We carried out overexpression experiments on members of the RBR loop which lead to loss of oscillations consistent with our predictions. These findings challenge the role of the RBR loop as a merely auxiliary loop and might change our view of the clock molecular circuitry and of the function of the nuclear receptors (REV-ERB and ROR) as a putative driving force of molecular oscillations.

## Author Summary

Most organisms have evolved an internal clock which allows them to anticipate and react to the light/dark daily rhythm and is able to generate oscillation with a circa 24 hour rhythm. A molecular network involving feedback loops is responsible for the rhythm generation. A large number of clock-controlled genes pass on time messages and control several biological processes. In spite of its medical importance (role in cancer, sleep disorders, diabetes and others) the mechanism of action of the circadian clock and the role of its constituent's feedback loops remains partially unknown. Using a mathematical model, we were able to bring insight in open circadian biology questions. Firstly, increasing the mRNA degradation rate of *Per* can contribute to increase or decrease of the period which might explain contradictory experimental findings. Secondly, our data points to a more relevant role of the ROR/*Bmal*/REV-ERB loop. In particular, that this loop can be an oscillator on its own. We provide experimental evidence that overexpression of members of the ROR/*Bmal*/REV-ERB lead to loss of *Bmal* reporter mRNA oscillations. The fact that REV-ERB and ROR are nuclear receptors and therefore important regulators in many cellular processes might have important implications for molecular biology and medicine.

## Introduction

Circadian rhythms can be found in most organisms, from bacteria to humans and are a fundamental property of living cells [1]. These endogenous rhythms provide a way to anticipate external cues and to adapt molecular and behavioural processes to specific day-times with the advantage of temporally separating incompatible metabolic processes [2].

At the core of the system is the circadian clock, a complex network of genes able to generate stable oscillations with a period of *circa* 24 hours. The circadian clock has been studied in detail in various organisms such as *cyanobacteria* [3], *Neurospora* [4], *Arabidopsis* [5], *Drosophila* [6] and mammals [7]. In mammals the main oscillator resides within the suprachiasmatic nucleus (SCN) and is directly entrained by light via the retinohypothalamic tract [8]. This central pacemaker in the SCN is formed by a set of roughly 20.000 neurons which produce rhythmic outputs and orchestrate local clocks in the brain and peripheral clocks throughout the body. Peripheral clocks in the liver, heart, kidney and skin are implicated in the regulation of local transcriptional activity. These can be synchronized also by external cues such as temperature and feeding schedules [9], [10].

Circadian clocks are evolutionarily conserved [6], [11] and designed to maintain an overall optimal organism activity. The internal pacemaker is responsible for the regulation of several biological processes at the cellular level. Such processes include sleep-awake cycles, memory consolidation [12], [13], metabolism of glucose, lipids and drugs [14], [15], bone formation [16], hormone regulation, immunity [17], the timing of cell division cycle and the physiological rhythms such as heart rate, blood pressure and body temperature [18]. Malfunctions of the circadian clock have been reported to be involved in many diseases and disorders such as susceptibility to cancer [19], familial sleep disorders (FASPS) [20], bipolar disorder, sleep problems in the elderly, seasonal affective disorders (SAD) [21], [22], diabetes [23] and obesity [24].

The daily regulation of molecular processes has severe consequences on therapy optimization and timing of drug intake, with the potential of minimizing toxicity and increasing treatment efficacy in complex diseases such as cancer [25]. Therefore, many efforts have been made to identify and understand the molecular circuitry of the clock and its role in disease and therapy [26], [27].

The mammalian molecular clock network is constituted by at least two large interconnected feedback loops which are able to generate approximately 24 hour rhythms [28], [29]. The heterodimer complex, CLOCK/BMAL, formed by the product of the genes circadian locomotor output cycles kaput (*Clock*) and brain and muscle aryl hydrocarbon receptor nuclear translocator like – Arntl (*Bmal*) represents the central node in the network and the transcription initiator of the feedback loops.

CLOCK/BMAL binds to E-box *cis*-elements in the promoter regions of target genes *Period* homolog 1, 2 and 3 genes (*Per1*, *Per2*, *Per3*), *Cryptochrome* genes (*Cry1*, *Cry2*), retinoic acid-related orphan receptor (*Rora*, *Rorb*, *Rorc*) and *Rev-Erb* nuclear orphan receptor (*Rev-Erbα*, *Rev-Erbβ*) to activate their transcription [7], [30].

The negative PER/CRY (PC) feedback loop is commonly seen as the primary generator of the circadian rhythm [31]. Transcription of *Per*s and *Cry*s is initiated during the circadian day. Aided by post translational modifications, PER and CRY proteins enter the nucleus, probably as a multimeric complex (PER/CRY) [32], and inhibit CLOCK/BMAL-mediated transcription after a certain delay [31]. The PER/CRY complex is degraded during the night, which releases its inhibitory action on CLOCK/BMAL and allows a new cycle of transcription to take place.

**Figure 1:**
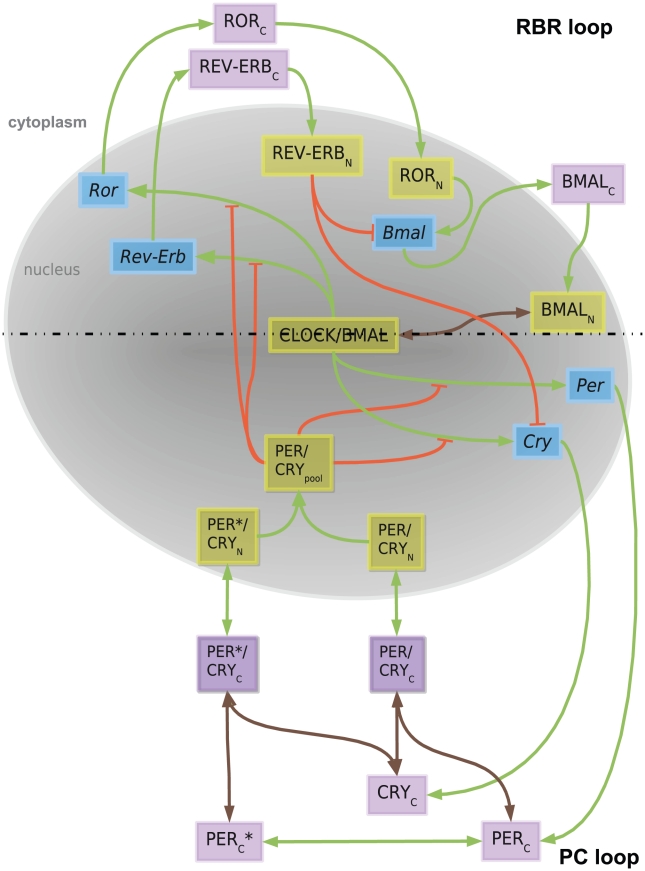
A model for the mammalian circadian clock. The model comprises two major compartments, the nucleus (light grey) and the cytoplasm. There are 20 species including 5 genes (highlighted in blue boxes), their corresponding cytoplasmic proteins and cytoplasmic protein complexes (indexed “C” and highlighted in violet boxes) and nuclear proteins and nuclear protein complexes (indexed “N” and highlighted in yellow boxes). Dead-end orange lines represent transcription inhibition reactions brown lines represent complex formation/dissociation reactions and green arrows show other reactions (transcription, translation, import/export, phosphorylation/dephosphoryplation). The dashed horizontal line visually divides the model into two large subunits: the RBR loop and the PC loop.

The ROR/*Bmal*/REV-ERB (RBR) feedback loop is usually seen as adding robustness to the system [31]. *Ror*s and *Rev-Erb*s are transcribed during the subjective day. Following translation, ROR and REV-ERB proteins compete for ROR regulatory element (RRE) binding sites in the promoter region of *Bmal* and regulate its transcription. ROR acts as an activator of *Bmal* and REV-ERB as an inhibitor which results in a fine-tuning of *Bmal* transcription [33]. Once in the nucleus the BMAL proteins form heterodimer complexes with CLOCK and initiate transcription of target genes (Figure 1).

Minimal models such as the Goodwin oscillator were the first to describe a negative feedback oscillator involving three components [34], [35]. Several kinetic models of the mammalian circadian clock have been subsequently developed [36], [37], [38], [39]. Early models miss essential components such as the nuclear receptor ROR or posttranslational modifications. Other models are rather large and thus the estimation of kinetic parameters becomes exceedingly difficult. Still, many issues regarding the clock remain unknown or not completely understood.

We propose here a single cell model for the mammalian mouse clock of intermediate complexity but containing the most essential biologically relevant processes. Our model allows an independent study of the two loops (PC and RBR). It is biologically comprehensive, emphasizes a parameterization based on biochemical observables, and reflects the current state of research. Although much is known about the circadian clock network, the kinetics of many reactions is not known which makes the parameterization process complex. We have explored known phases and amplitudes among the model components and made use of control theory's principles [40], to obtain estimations for many of the unknown parameters. The resulting model is tested using published data on genome-wide RNAi experiments [41], [42] and transcriptional inhibition data [43].

Our model was applied to address open questions in circadian rhythm biology: firstly, what are the possible reasons for the observed two-loop design? Mathematically, one negative feedback loop with a time delay would be enough to generate stable oscillations. There is evidence from published data showing that overexpression of components of the PC loop does not destroy oscillations [44], [45] which together with remarkable phenotypic effects for members of the RBR loop [46] motivated us to investigate the role of the RBR loop in detail. Secondly, how does degradation kinetics affect the period? We emphasize that such questions cannot be answered intuitively but require quantitative models. The period of the system depends on the timing of gene expression, accumulation and decay, and since clock protein degradation can influence all these processes, intuitive predictions are difficult.

Our simulations show that faster degradation of clock proteins can indeed lead to shorter and longer periods under certain circumstances. In addition, our model predicted that overexpression of members of the RBR loop would lead to damped or even to the loss of oscillations. We could verify these predictions experimentally by constitutively overexpressing *Ror* and *Rev-Erb* RNAs in U2OS cells.

Our study represents a step forward towards a fully parameterized model holding significant predictive value. Moreover, this work brings valuable insights into circadian clock biology and helps to understand apparently contradictory results.

## Results

### Characterisation of the gene network – Model design

We developed a model for the mammalian circadian clock, which allows the study of the two main feedback loops: ROR/*Bmal*/REV-ERB (RBR) and PER/CRY loop (PC). The model can also be used to study mechanisms critical for the tuning of the circadian system including transcription, translation, import/export, degradation and phosphorylation. We decided to focus on the main pacemaker in the SCN which is assumed to be responsible for the synchronization of the circadian system. Furthermore, the SCN clock might be accountable for general malfunctions and consequent failure of peripheral clocks function, leading to the disruption of normal rhythms [19], [20], [25].

The model was designed based on an extensive literature search and accounts for the available experimental facts (Dataset S1) of the mouse core clock, but is still small enough to allow a systematic parameter determination. For our data collection we gathered available expression data for phases and amplitudes for all the components of the system, regarding the SCN. In order to compare amplitudes of different components found in the literature we normalized the expression level of each component to its mean value. This procedure enables the simulation of expression profiles that oscillate around a base line of 1, for all variables, facilitating the comparison among them. With the developed model we were able to investigate the effects of transcription and degradation on the period of the system and to shed light in a putative role of a two-loop design.

The model contains 19 dynamic variables distributed along two main feedback loops that might be virtually separated (Figure 1, dashed line). Interlocked feedback loops were also reported for N*eurospora* [47], *Drosophila melanogaster* [48] and *Arabidopsis thaliana* [49].

In our model we refer to gene family groups, or gene entities: *Per* (*Per1,2,3*) [50]; *Cry* (*Cry1,2*) [51]; *Ror* (*Rora,b,c*) [52], [53]; *Rev-Erb* (*Rev-Erbα,β*) [54], [55]; *Bmal* (*Bmal1,2*) [56]. The same principle applies to the proteins and respective protein complexes, represented in Figure 1. The central component, CLOCK/BMAL complex, binds to the promoter regions of clock genes (*Rev-Erb*, *Ror*, *Per*, *Cry*) activating their transcription [57], [58]. Transcription is controlled by PER/CRY (PER/CRY_pool_) which possesses an inhibitory effect [59]. In our model the complex represents the pool of all possible PER/CRY complexes present in the nucleus including phosphorylated and unphosphorylated species. We consider in the model the effect of PER/CRY as a transcription inhibitor, regardless of the detailed mechanisms [60], [61].

#### The ROR/*Bmal*/REV (RBR) loop

The clock genes *Rev-Erb* and *Ror* are transcribed and the messages are exported to the cytoplasm for translation. The newly translated proteins, ROR_C_ (RORa,b,c cytoplasmic proteins), REV-ERB_C_, (REV-ERBα,β cytoplasmic proteins) of the RBR loop are then imported to the nucleus. Once in the nucleus, ROR_N_ (RORa,b,c nuclear proteins) and REV-ERB_N_ (REV-ERBα,β nuclear proteins) participate in the regulation of *Bmal* as activator and inhibitor, respectively [33]. *Bmal* is translated in the cytoplasm, giving rise to BMAL_C_ (BMAL cytoplasmic protein) which is imported to the nucleus as BMAL_N_ (BMAL1,2 nuclear protein) and there binds CLOCK forming the CLOCK/BMAL complex.

The formation of the CLOCK/BMAL complex is reversible (Figure 1, double-end connector). A peculiarity of the design of the RBR loop is its capability of influencing the PER/CRY loop via the inhibition of REV-ERB_N_ on *Cry* [46]. Liu *et al*. have shown that the levels of *Cry1* decrease upon *Rev-Erb* overexpression in *Bmal1* knockout mice. This data could be reproduced *in silico* by our simulations (data not shown). The cross connection with REV-ERB [59] reinforces the interaction of the two loops and allows fine-tuning of the regulation of *Cry*.

#### The PER/CRY (PC) loop

Within the PC loop, represented on the lower half of the Figure 1, the newly transcribed genes *Per* and *Cry* are translated in the cytoplasm as PER_C_ (PER cytoplasmic protein) and CRY_C_ (CRY cytoplasmic protein). PER_C_ is phosphorylated reversibly giving rise to PER_C_*. We consider PER_C_ to be a *hypophosphorylated* (since a completely nonphosphorylated PER would presumably not be able to enter the nucleus). For example, in patients suffering from familial advanced sleep phase syndrome (FASPS) PER2 is hypophosphorylated but can still enter the nucleus and repress CLOCK/BMAL1 transactivation [20], [62], [63]. It is known that other phosphorylation steps can occur affecting both CRY_C_ [64] and the phosphorylated form of PER_C_ [20], [65] as well as CLOCK [66], BMAL [67] and REV-ERB [68] but these are not considered in the present model.

The three proteins, PER_C_, CRY_C_ and PER_C_* form complexes in a reversible manner, and these complexes PER*/CRY_C_, PER/CRY_C_ can be imported to the nucleus [29], [32], [69], [70]. Nuclear shuttling is allowed in our system and explicitly modelled for PER/CRY complexes. For the other variables the import rates to the nucleus considered in the model might reflect shuttling as well, but this would be at this point speculative and can only be addressed when more data for those variables is available. Nuclear accumulation of the proteins is mainly achieved by using a nuclear import rate higher than the nuclear export rate. The nuclear complexes PER/CRY_N_ and PER^*^/CRY_N_ are pooled together as a unique virtual PER/CRY_pool_ complex which acts as an inhibitor and closes the loop. All components of the system, both cytoplasmic and nuclear, are subject to degradation [71]. Since the kinetics of degradation in the system is not known, we assumed linear rate laws [72]. A table containing all parameters used, together with the ordinary differential equations (ODEs) that describe the network, is provided in Text S1.

Three major levels of regulation can be seen in the system. The first concerns protein-DNA interactions, where regulation is achieved via activation/inhibition of *Bmal* transcription. A second regulation step is found at the protein-protein interaction level where the repressor complex, PER/CRY, regulates the system by blocking CLOCK/BMAL-mediated transcription. Finally, RNA and protein stability plays a major role in the tuning of the system and is implicated in the regulation of phases of all gene entities. Posttranslational modifications, such as phosphorylation [73], also contribute to the delay which is necessary to generate a *circa* 24 hour period.

### Analysis and optimization of the system

The circadian core clock network (Figure 1) can be translated into a system of 19 ordinary differential equations (ODEs) with 71 parameters given in Text S1. The system of equations was assembled using mostly the law of mass action [74] and linear degradation kinetics. A Michaelis-Menten degradation kinetics could be used as done in other models allowing smaller Hill coefficients [36], [75]. However, such kinetic laws need more parameters, therefore, we decided to use linear laws in order not to increase the complexity of the model.

Nonlinearities were introduced to describe transcription reactions by means of Michaelis-Menten [76] kinetics and Hill functions [77]. Many parameters could be retrieved from the literature and others were estimated based on known phases and amplitudes using LTI (linear-time-invariant) systems theory (Text S2).

The LTI system theory is often used in electrical engineering, signal processing and control theory. It implies the linearization of the system. Therefore, we created a linear ODE version of the network and applied LTI to our system which allowed a partial determination of the parameters. Linear models allow the analytical calculation of amplitudes and phases as functions of the parameters [40]. Each feedback loop was transformed into a linear open loop system which was then closed, re-establishing the feedback. The parameters were optimized in order to achieve the optimal amplitude and phase-relations. In a subsequent step values for the corresponding parameters of the nonlinear system were determined using a Taylor expansion. After closing the loop the parameters were finally optimized to fine-tune the model.

We based our calculations using key biological assumptions relevant for the mammalian circadian oscillator, such as a period of about 23.5 hours and measured phase/amplitude relations between the components of the model. The parameter estimation procedure is described in detail in Text S2. Still 11 of 71 parameters remain free and their values were adapted to fine-tune the phase and amplitude relations.

**Figure 2:**
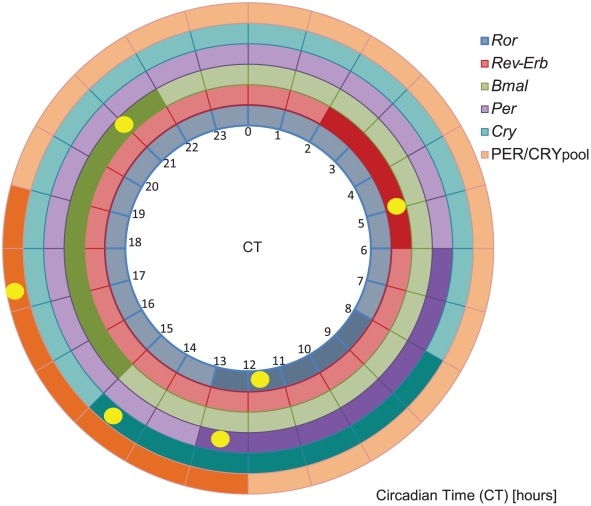
In *silico* expression data fits known experimental data. The circular graphic shows expression data for 6 variables present in the model: 5 RNAs (*Ror*-blue; *Rev-Erb*-red; *Bmal*-green; *Per*-violet; *Cry*-turquoise) and the PER/CRY nuclear pool (orange). Each variable is represented by one ring. The dark colour tone marks the published time interval corresponding to the highest expression level of the given variable. The full yellow circle indicates the peak of expression in our *in silico* experiments. Correspondent circadian times (CT) in hours are given.

The resulting model generates oscillations with a period of 23.5 hours and is able to simulate RNA and protein peaks of expression in the range of the ones found in the literature (Figure 2). The circular graphic shows a comparison between the *in silico* peaks of expression and the corresponding experimental intervals found in the literature. Represented are 4 mRNA sets (*Ror*, *Rev-Erb*, *Bmal*, *Per*, *Cry*) and the nuclear protein complex PER/CRY_pool_ (PER/CRY nuclear pool), covering both parts of the model (RBR loop and PC loop). *Bmal* mRNA reaches its maximum of expression in the early night. After translation the protein participates in the activation of its target genes in the nucleus. *Rev-Erb* has its highest expression in the early morning, followed by *Ror* and *Per* and finally *Cry* in the late morning/early afternoon. The heterodimer complex PER/CRY reaches its nuclear expression peak in the late afternoon closing the cycle.

### In *silico* data are consistent with experimental data

We have tested the predictive capability of the model by comparing results of our simulations with mutation data from knockout mice (Dataset S1) and RNAi data from U-2OS cells [41], [42]. The resulting period of the oscillations was analyzed and given as an output of the simulations. As shown by Brown *et al*. [78] single cell data might reflect behavioural phenotypes. Thus predictions from our single cell model can be compared with observations from animal mutational phenotypes.

The experimental variability between animal model and cell line data, or even the same system but different publications is higher than the discrepancy between *in silico* and experimental data (Table 1). This might be due to the fact that the clock system is extremely complex, eventually with more redundancy and further parallel sub-pathways than established so far. It would be conceivable that more feedback loops involving the clock and interconnected networks [79] exist and explain the variability of the phenotypes. Moreover, our control analysis indicates (see Table S1) that variability of some parameters such as degradation rates might be accountable for phenotypic differences between animals and cell lines.

**Table 1:** Effect of clock gene dosage on RNA circadian period-comparison of *in silico* with experimental data.

Gene	Mutation phenotype	*in silico* data-mutants	siRNA data [42]	siRNA data [43]	*in silico* data						in *silico* data-siRNA	
	animal model – mouse	transcription rate (−90%)	U-2 OS cells	U-2 OS cells	rna degradation rate (+10%)	translation rate (−10%)	rna degradation rate (+50%)	translation rate (−50%)	rna degradation rate (+70%)	translation rate (−70%)	rna degradation rate (2-fold increase)	translation rate (2-fold decrease)
***Clock ^Δ19/Δ19^***	**+**	**+**	**AR**	**+**	**nd**	**nd**	**nd**	**nd**	**nd**	**nd**	**+**	
***Clock^−/−^***	**-**											
***Bmal1***	**AR**	**AR**	**AR**	**+/−**	**WT**	**WT**	**AR**	**AR**	**AR**	**AR**	**- then AR**	**- then AR**
***Bmal2***	**nd**		**WT**	**nd**								
***Per1***	**- then AR**	**AR**	**AR**	**+**	**WT**	**+**	**+**	**+**	**+**	**+**	**+**	**+**
***Per2***	**- then AR**		**+**	**+**								
***Per3***	**−**		**−**	**−**								
***Per1+Per3***	**- then AR**		**nd**	**nd**								
***Per2+Per3***	**- then AR**		**nd**	**nd**								
***Per1+Per2***	**AR**		**nd**	**nd**								
***Cry1***	**−**	**+**	**−**	**+**	**WT**	**WT**	**WT**	**+**	**+**	**+**	**WT**	**+**
***Cry2***	**+**		**+**	**+**								
***Cry1+Cry2***	**AR**		**AR**	**nd**								
***Rev-erbα***	**−**	**AR**	**+**	**+/WT**	**WT**	**WT**	**AR**	**AR**	**AR**	**AR**	**- then AR**	**- then AR**
***Rev-erbβ***	**nd**		**WT**	**+/−**								
***Rorα***	**−**	**- then AR**	**WT**	**−/WT**	**−**	**WT**	**AR**	**AR**	**AR**	**AR**	**- then AR**	**- then AR**
***Rorβ***	**+**		**WT**	**−/WT**								
***Rorγ***	**WT**		**WT**	**nd**								
***CK1ε^tau^***	**−**	**+**	**+**	**nd**	**nd**	**nd**	**nd**	**nd**	**nd**	**nd**	**+**	
***CK1ε^−/−^***	**+**											

WT, wild type; +, period increase; +/WT, experimental replicates leading to different results; +/−, experimental replicates leading to different results; −, period decrease; −/WT, experimental replicates leading to different results; AR, arrhythmic phenotype; − then AR, decrease in the period followed by arrhythmic phenotype; nd, not defined.

#### In *silico* knockouts produce similar phenotypes to KO mice

In order to compare model predictions to knock out experimental data, we carried out a series of simulations where we decreased the transcription for each gene entity in the model by 90% (decreasing the correspondent transcription rates *V_max_* by 90%) and quantified the corresponding effect on the system. The results of the simulations together with a compilation of similar experimental published data are presented qualitatively in Table 1. The table shows a compilation of published data regarding mutational phenotype, column 2 (data in Dataset S1) and siRNA (columns 4 and 5) experiments [41], [42]. *In silico* mutational data is obtained by a 90% decrease in the transcription rate of the corresponding gene (column 3). Simulations of siRNA experiments were carried out by increasing the degradation rate for the corresponding RNA. A step wise increase is shown, simulating an increasing RNAi efficiency (columns 6, 8, and 10). The direct effect of siRNA experiments at the protein level was also simulated by decreasing values of the translation rate for the respective RNA (columns 7, 9 and 11). In the last 2 columns we present *in silico* data for siRNA simulations where the transient region of the simulation output is analysed, such results correspond to the immediate effect of the perturbation on the system.

We consider a plus (+) for an increase of the period of more than 1% of the wild type value (WT), and a minus (−) for a decrease of more than 1%. Variations between −1% and +1% are not considered as significant and are labelled as WT. Should the oscillations become damped or vanish after the perturbation, we consider the system to be arrhythmic (AR). Detailed quantitative results from the simulations can be found in Dataset S2.

It is essential to bear in mind that in the model groups of genes are represented, and not individual genes (e.g. *Per* stands for *Per1,2,3*). A 100% reduction of transcription would disable any possible redundancy and/or compensation among family members which was reported to happen in the circadian clock [41]. Taken this into account, our knock out simulations might be closer to multiple KO phenotypes effects. As an example, the double KO of *Rev-Erbα* and *Rev-Erbβ*, has been described as leading to loss of oscillations [46] as we could also predict from the model simulations (Table 1).

#### *In silico* reproduction of RNAi experiments

To simulate the effect of RNAi experiments we analysed the effect of interference at two levels: a) at the RNA level by perturbing the RNA degradation rate and b) at the protein level analysing the effects of disturbing the translation rate. Therefore, we applied independently an increasing RNA degradation rate and a decreasing translation rate. Both methods gave similar results and verified published data (Table 1). For a detailed analysis we have primarily analysed the transient region of the oscillations, which is usually discarded in simulations, since the system is still evolving at that point. However, RNAi assays induce a transient transformation of the system [42], and we therefore believe it to be relevant to consider transients. This data is given in the last two columns of Table 1 and it reinforces the previous results. Cell lines data exist for several clock mutants [41] that confirm the animal phenotypes compiled in Table 1.

### A coupled two-loop system

**Figure 3:**
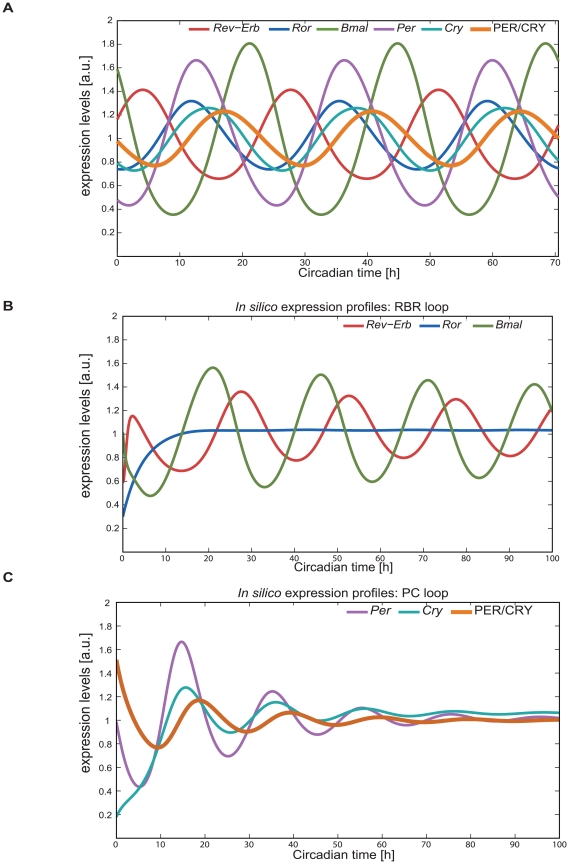
The mammalian circadian clock can be represented by a merged two-loop system. A) *In silico* expression profiles show robust oscillations with a period of 23.5 hours can be obtained with the model. *In silico* expression data for phases and amplitudes fit known published experimental data. Elements of the RBR loop (*Rev-Erb*, *Ror*, *Bmal*) and from the PC loop (*Per*, *Cry*, PER/CRY_pool_) are represented. B) *In silico* expression profiles for the RBR loop. The RBR loop is a low amplitude oscillator given a constitutive PC loop. This loop is able to oscillate with smaller amplitude and larger period then the full clock model. The oscillatory effect of the PER/CRY pool is replaced by its mean value (PC = 1.71). The behaviour of the system for different mean values of PC is shown in Supplementary Figure S2. C) The PC loop is a damped oscillator (for our default parameters) given a constitutive RBR loop. The connection to the RBR loop is replaced by a constitutive CLOCK/BMAL and a constitutive REV-ERB nuclear (each variable is replaced by its mean value, *x1* = 1.7 and *x5* = 2.4).

We used the optimized model to analyse the oscillatory potential of each loop as an independent oscillator [80]. Our complete model shows oscillatory expression patterns with a period of 23.5 hours for all components (Figure 3A) and simulates the phase differences and relative amplitudes found in the literature (for comparison see Dataset S1).

Analysing the delays between the different gene species involved in the model (Dataset S1) large delays in the RBR loop can be found. This suggests that this loop could act as an oscillator, also when decoupled from the system. We have therefore hypothesized that the RBR loop should be able to oscillate in the absence of an oscillatory driving force. To test this hypothesis, we replaced the variable PER/CRY_pool_ by its mean value (Text S1, *PC* = 1.71), creating a constitutive inhibitor. We further wished to analyse the robustness of the model regarding PC and carried out a set of 6 *in silico* experiments were the PC wild type value is perturbed (*PC_WT_* = 1.7) to +/−10%, +/−20%, +/−50% (Supplementary Figure 2). As shown in the figure the oscillations are preserved also under these conditions. This RBR subsystem is a low amplitude oscillator, with a 25.1 hours period (Figure 3B). Interestingly the expression pattern of *Ror* RNA is almost constant which is consistent with the fact that the inhibitor *Rev-Erb* might be the driving force of the RBR loop. We aimed to further investigate the independent role of the PC loop. Therefore, we simulated the decoupling of the PC loop by replacing CLOCK/BMAL and REV-ERB_N_ by their mean values (Text S1, *x1* = 1.7; *x5* = 2.4) generating a constitutive inhibitor and activator respectively. The PC sub-system is a damped oscillator (Figure 3C) with a shorter period (20.7) then the coupled oscillator system.

**Figure 4:**
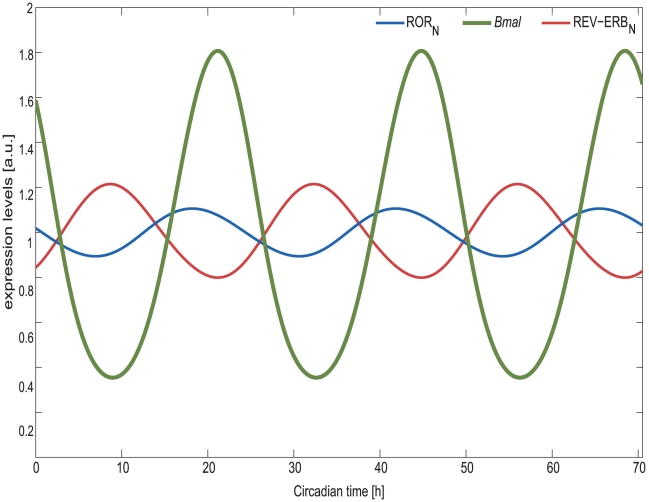
*Bmal* is regulated by the antagonistic action of REV-ERB and ROR. Represented are *in silico* expression profiles for the nuclear protein REV-ERB_N_ and ROR_N_, and for *Bmal* RNA. The nuclear proteins ROR_N_ (red) and REV-ERB_N_ (blue) recognize and compete for the *cis*-regulatory elements in the *Bmal* (green) promoter region to act, respectively, as positive and negative drivers of *Bmal* expression.

A negative feedback can induce circadian oscillations if the delay is at least 6 hours [81]. The observed delays between *Bmal* transcription and its inhibition via REV-ERB_N_ exceed 6 hours (Figure 4). Thus it is conceivable that RBR loop is indeed an oscillator on its own as indicated by our findings.

### Competition for Rore elements at the promoter region: fine tuning of *Bmal*

The nuclear receptors ROR and REV-ERB have been reported to control *Bmal* expression by competing for RORE elements in the promoter region of the gene and exert an opposite effect on the regulation of *Bmal*. Our model can simulate the pattern of ROR_N_ and REV-ERB_N_ protein expression and its correlation with *Bmal* RNA expression, thereby illustrating the mechanism of *Bmal* regulation (Figure 4). The expression curve of both proteins is almost in anti-phase, which was obtained as a result of imposing a specific amplitude and phase for *Bmal* RNA. From the theoretical standpoint REV-ERB and ROR need to have opposite expression values in order to induce robust transcription of *Bmal* with a concentration and time for peak of expression according to published data. When the inhibitor REV-ERB_N_ is at its maximum *Bmal* reaches its minimum expression value and ROR_N_ starts increasing its production. Some hours later ROR_N_ reaches its maximum and REV-ERB_N_ reaches its minimum level, leading to a peak of *Bmal* expression. Both REV-ERB_N_ and ROR_N_ act antagonistically to enhance *Bmal* oscillations, which will then regulate the transcription of the genes in the model. These observations are in agreement with experimental findings [33].

Is rhythmic activation of REV-ERB and ROR necessary for *Bmal* oscillation? To answer this question we carried out further simulations in which we replaced the activator and inhibitor by constitutive ones with corresponding mean value. When REV-ERB_N_ is replaced by its mean value (Text S1, *x5* = 2.4) the oscillations of all components in our network are lost. Interestingly, if we increase the concentration of the constitutive inhibitor we recover *Bmal* oscillations. This could be related to the fact that REV-ERB_N_ acts as an inhibitor of *Cry* as well. Increasing REV-ERB_N_ induces an inhibitory effect on *Cry* transcription leading to a decrease of the PER/CRY_pool_ and therefore to a decrease of the inhibition on CLOCK/BMAL. This leads to an increase of ROR_N_ and a consequent recovery of *Bmal* oscillations. If on the other hand, ROR_N_ is replaced by its corresponding mean value (Text S1, *x6* = 5.8), *Bmal* still oscillates but with smaller amplitude (data not shown). These results indicate that the amplitude and phase relation between activator and inhibitor is crucial to generate a proper oscillating *Bmal* with the correct phase and amplitude. Taken together, results from our simulations point to a more important role of the RBR loop on the clock system, than previously assumed.

### Effect of transcription on the period

Published data indicate an influence of the transcription rate on the period of the system [43]. We have addressed this question by perturbing the transcription of each of the five gene entities present in the model and measured the resulting period. A detailed table with all data corresponding to a gradient of the transcription rate from a 10 fold decrease to a 10 fold increase to the wild type is given as Table 3 in Dataset S2.

Analysing the effect of an overall increase in the transcription rate on the system, we observe a direct correlation to the period. As a response to an overall transcription increase, we obtain a longer period revealing a delay of the clock (period measured for the *in silico* reporter gene *Bmal*). On the other hand by decreasing the overall transcription rates we obtain a shorter period which accounts for a hastening of circadian oscillations as reported by Dibner *et al*. [43]. Interestingly, we observe that only perturbations on *Cry* transcription do not lead to loss of oscillations. The same type of perturbation in the remaining 4 gene entities, leads to loss of oscillations. Moreover, for a decrease of the transcription rate of *Per* and *Cry* an increase of the period is observed pointing to their role as inhibitors. The same can be seen when decreasing the rate of transcription for *Rev-Erb*. This effect is opposite for the activators *Ror* and *Bmal* where an increase of the respective transcription rates leads to an increase of the period of the system.

### Effect of degradation on the period

The effects of the degradation of *Per* on the period are very complex and not yet clarified [82]. This aspect can be exemplified by the following question: Is a faster degradation of a clock element, such as *Per2*, leading to a shortening or lengthening of the period? Degradation rates are intimately related to the effective delay [65], [83], [84] and consequently one might expect that faster degradation leads to a shorter delay and, subsequently, to a shorter period. This is indeed observed in a cellular model of the FASPS disorder [20]. On the other hand, fast degradation might slow down the nuclear accumulation of the inhibitory PER/CRY complexes leading to a prolonged period. This expectation sounds reasonable as well, thus, intuition alone leads to contradictory predictions and therefore detailed quantitative considerations are required to answer the question raised above.

**Figure 5:**
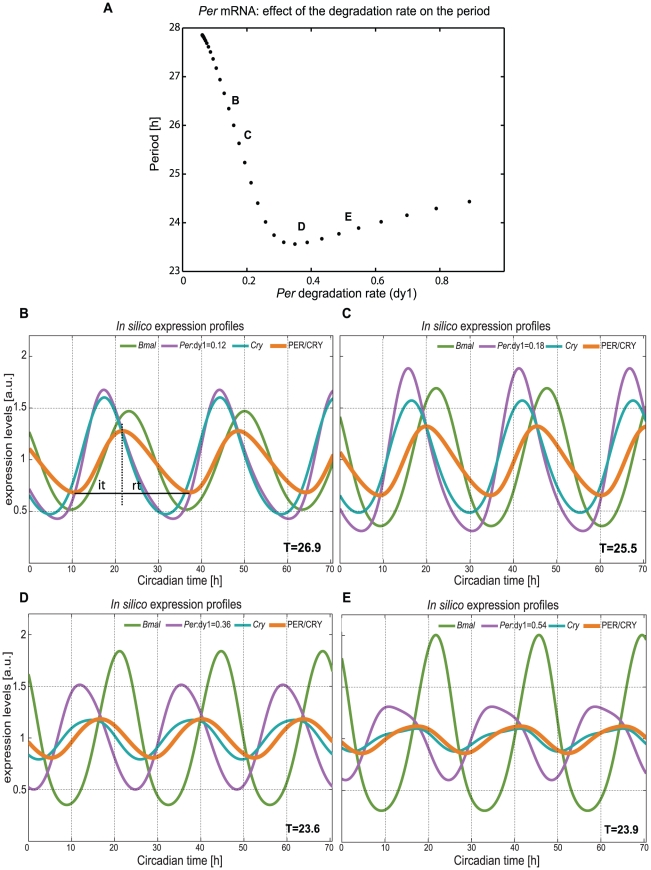
Degradation control can increase and decrease the period. A non-monotonic behaviour can be seen for *Per* RNA when a gradient of the degradation rate is applied to the system. The graphic represents the period as a function of the degradation rate. We marked 2 points (B, C) in the decreasing period region of the graphic and 2 points (D, E) in the increasing period region. (**B**,** C**) The period decreases with increasing degradation rate of *Per* RNA (*dy1*). (**D**,** F**) The period increases with increasing degradation rate of *Per* RNA (*dy1*). The wild type scenario is shown in Figure 3 A.

Mutational phenotypes of *Per* genes indicate in most cases period shortening or arrhythmic phenotype (Table 1). However, simulation data in Table 1 shows also an increase of the period, with increasing degradation rate. We found these observations remarkable and used the model to find a possible explanation. Our results are quite surprising as can be seen in Figure 5A. We analysed in detail the behaviour of *Per* when continuously changing its RNA degradation rate (Text S1, *dy1*) from 0 to 1 (3.3 fold increase of the WT value). *Per* has a non-monotonic behaviour regarding the degradation rate. This could explain why we see an increase in the period when increasing the degradation rate (Table1) and on the contrary there are published phenotypes showing a decrease.

Figures 5B–5D show simulated time-series of clock genes which can be analysed to understand the underlying mechanisms of non-monotonic period changes. If we choose a value for the degradation rate within the first part of the graphic (marked points B, C) then a decrease of the period with increasing degradation rate would also be seen (Figure 5A). In the second part (points D, E) an increase of the period with the degradation rate is observed. As the degradation rate increases, the system moves from a scenario where the amplitudes of *Per* and *Bmal* are small and *Per* is in phase with *Cry* (Figure 5B) to another where *Per* and *Bmal* amplitudes are larger (Figure 5C). Analyzing the profile of the inhibitor (PER/CRY) it is visible that the shape of the wave varies considerably. The time needed for PER/CRY to reach its inhibitory peak of action (inhibition time, *it*) and the time needed for it to reach the trough of expression (release time, *rt*) is different for the 4 points marked (Figure 5A). This might account for the variation in the period. Therefore, we extracted the inhibition times and release times for Figure 5B (*it* = 11 hours; *rt* = 16 hours) and Figure 5C (*it* = 10.5 hours; *rt* = 15 hours). The values measured (3.4% decrease in *it* and a 7.4% decrease in *rt*) together with a sharper peak of the inhibitor complex due to amplitude and phase changes of *Per* and *Cry* are correlated with a shorter period (Figure 5C). Due to the earlier phase of *Per* in Figure 5C compared to Figure 5B the release from inhibition is fastened.

In the second part of the graphic (Figure 5D, 5E) the opposite happens. The period increases with the increase of the degradation rate. The increase in the degradation rate leads to even larger phase shifts between *Per* and *Cry*. Following the same methodology, we measured the inhibition and release times for PER/CRY for Figure 5 D, (*it* = 11.5 hours; *rt* = 12.1 hours) and for Figure 5E (*it* = 12,9 hours; *rt* = 11 hours). These changes correspond to a 10,8% increase of the inhibition time and a 10% decrease of the release time leading to a 1.2% increase of the period. Long inhibition times might be correlated with an increase of the period. This intricate discussion of phase relationships and wave forms helps to understand seemingly counter-intuitive observations. Interestingly, non-monotonic dependencies were found also in much smaller models and with different kinetics than ours [85], [86].

One well studied effect of the degradation on the period of the system is a circadian disorder, FASPS [20], which was the first reported pathology to link known core clock genes to a human disorder. The disease results from a mutation in a casein kinase binding site which affects the phosphorylation of PER and therefore its degradation and results in a circadian oscillator with a shorter period.

We wished to analyse the possible cause for the period shortening knowing that PER's degradation is affected. One possible mechanism could be that FASP mutation reduces the nuclear retention of PER2 but the turnover is not affected [20]. In order to simulate this situation we have increased the export rate of the PER*/CRY nuclear complex to (*kex2* = 0.05) and we obtained a decrease in the amplitude of *Per* and a shorter period, as reported. An alternative situation could be that the turnover of nuclear PER is enhanced, as well as its degradation by the proteasome. This has been previously described as another form of FASPS from the reports on the *tau* mutation in CKIepsilon [87]. To simulate this hypothesis we have increased the degradation rate for the nuclear protein PER* (*dx2* = 0.1). As a result we obtain as well a shortening of the period and a decrease in the amplitude of *Per*.

These results show that our model can simulate this particular biological problem and is able to illustrate possible alternative scenarios. In this case the model indicates two possible perturbations in the PER* protein, either affecting the degradation rate or the import/export of the phosphorylated protein, both leading to the experimentally observed decrease in the period. With more precise experimental measurements regarding localization and degradation kinetics of *Per*, the model should be able to discriminate between the two scenarios.

### Computational predictions using overexpression experiments

**Figure 6:**
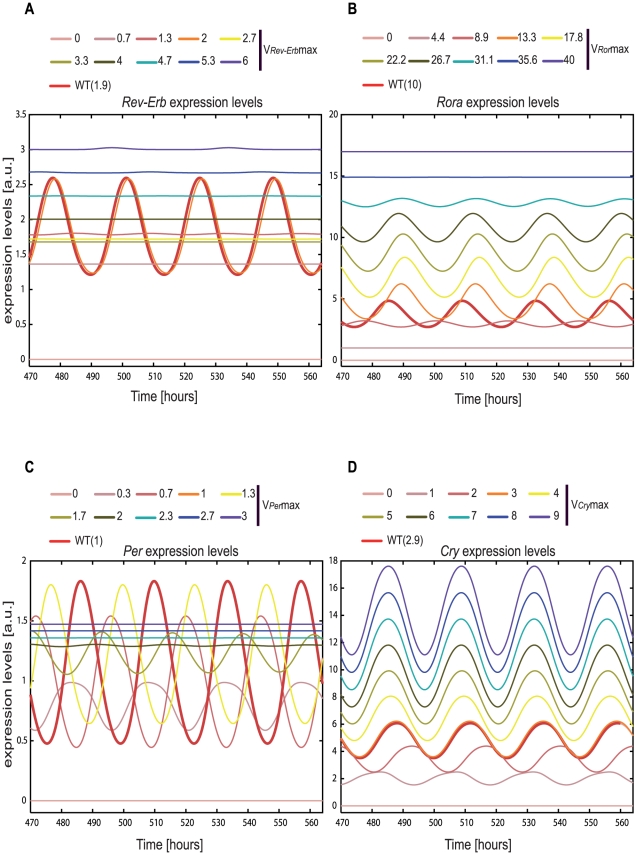
Increase in transcription in elements of both RBR and PC loops can destroy the circadian oscillator. (**A–D**) After a transient period of about 20 days we plotted the expression patterns for each of the four genes indicated. Skipping the transient region allows an enhancement of the effects on the system: damped oscillations give rise to flat expression profiles and small phase differences became better visible. Expression profiles for the indicated RNA are shown given different values of the respective RNA transcription rate. Transcription rates vary from 0 to approximately 3 times the value used in the model producing a total of 10 profiles. The profile curves are produced using Xppaut. We constrained the simulations to a fixed number of 9 steps. Each step corresponds to a specific value Vmax, equally distributed, within the range in which the parameter is allowed to vary. This procedure leads to a total of 10 expression profiles which together with the wild type profile generate the 11 profiles represented in each panel of the figure. The light pink line corresponds to a transcription rate value of 0 followed, in a step wise manner, by darker pink tones, orange, yellow, light and dark green, turquoise, blue and violet as a result of the highest value simulated for the transcription rate. The thick red line shows the expression profile corresponding to the transcription rate used in the wild type (WT) model.

Does a perturbation in the transcription of members of the RBR loop influence the system? To test this property of the upper RBR loop we perturbed the transcription of each gene entity independently and analysed its behaviour and influence on the system. We investigated the robustness of the 4 genes (*Rev-Erb*, *Ror*, *Per*, *Cry*) represented in the model. In other words we have tested if the oscillations in the expression of these genes are kept in regard to variations in the corresponding transcription rate (*V_max_*). For each of the genes the transcription rate was varied from 0 to about 3 times the original wild type value, *V_max_*(WT). This simulates two scenarios: a down regulation of gene expression, for values of *V_max_* lower than *V_max_*(WT); an increase in transcription of the RNA, for values of *V_max_* above *V_max_*(WT). The results from these simulations are displayed in the form of “rainbow-plots” (Figure 6). These plots provide similar information as bifurcation diagrams [88] with the benefit of allowing the simultaneous visualization of the gene expression dynamics. The rainbow plots allow the detection of sudden qualitative changes in dynamical behaviour of the system, upon small changes of the parameter analysed. We aimed to study the long term effect of the perturbations and hence have simulated 24 days and analysed the last 4 days (Figure 6). For all 4 gene entities there is an optimal parameter range, around *V_max_*(WT) value which allows the generation of oscillations with the desired phase and amplitude. Furthermore, for *Rev-Erb*, *Ror* and *Per* there is a defined optimal region where the system is able to oscillate, outside this region no oscillations are visible. Interestingly, *Cry* seems to be very resilient to perturbations. This could be related to the fact that this gene has two inhibitory mechanisms: PER/CRY and REV-ERB_N_ which together could compensate for the increase in transcription levels.

### The two-loop design principle

**Figure 7:**
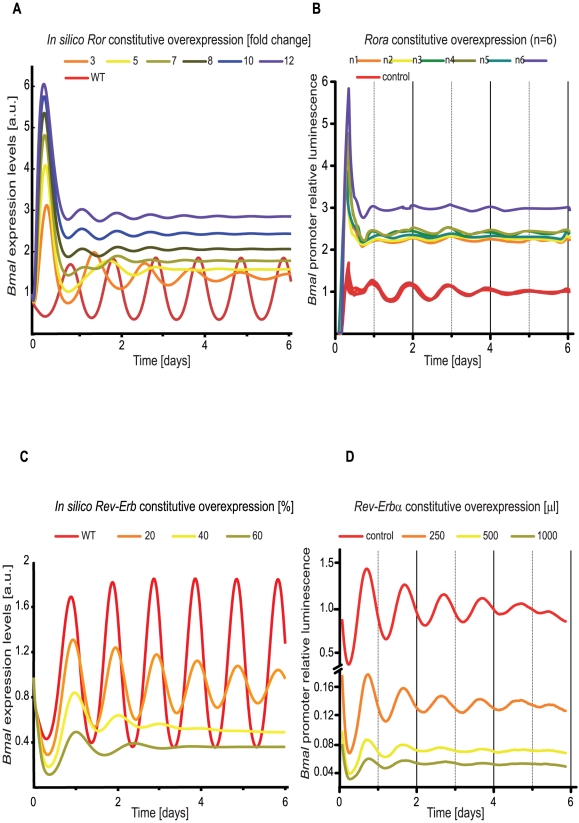
Experimental data for *Rora* and *Rev-Erbα* overexpression verify model predictions. The graphics show *Bmal* expression levels upon constitutive *in silico* and *in vitro* overexpression of *Ror* and *Rev-Erb*. (**A**) *In silico* overexpression of *Ror*. A constitutive exogenous *Ror* RNA is added to the system in increasing amounts. The ratio between exogenous constitutive *Ror* RNA and the mean value of the endogenous one is given. The wild type is shown in red. A gradient in fold change between exogenous RNA and the endogenous WT is shown from 3 to a maximum of 12 as indicated in the figure. (**B**,** D**) Human U-2 OS cells harbouring a *Bmal1*-luciferase reporter were lentivirally transduced with GFP control (red), or Rora (orange to blue) (B), or 250, 500 and 1000 µl lentiviral supernatant of Rev-Erbα overexpression plasmid. Cells were synchronized by a single pulse of dexamethasone and luciferase activity was monitored for several days. Depicted are de-trended data of biological replicas ((B) n = 6) and technical replicates ((D) n = 4) for each condition according to the fold difference in luciferase signal intensity of the reporter in *Rora* or *Rev-Erbα* overexpressing cells relative to GFP controls from the raw data. (**C**) *In silico* overexpression of *Rev-Erb*. A constitutive exogenous *Rev-Erb* is added to the system, the amount of endogenous RNA is given as a percentage of the endogenous wild type. The wild type is show in red and the increasing amount of constitutive exogenous RNA is show from 20 to 60% increase to the WT, as indicated in the figure. Overexpression data on *Rev-Erb* was previously reported as repressing Bmal1 as well [93].

We aimed to explore the role of the RBR loop in more detail and therefore overexpressed *in silico* both *Ror* and *Rev-Erb*. To attain such a perturbation we added a constant RNA, to the endogenous one, for both gene entities independently (Text S1). The constitutive exogenous RNA was taken together with the endogenous one and used for the subsequent protein production. As an output we analysed *Bmal* patterns of expression. We took care to run our simulations in an *in silico* set up which would correspond to a real experimental set up, and therefore examined the transient region of the simulations. The result of the simulations for 6 days is given in Figure 7. Our predictions show that *Bmal* magnitude increases upon *Ror* constitutive overexpression but the oscillations are lost after 6 days (Figure 7A). *Rev-Erb* constitutive overexpression leads to a decrease of *Bmal* magnitude. Similarly to what happens with *Ror*, the oscillations are also damped and lost after 6 days (Figure 7C).

To experimentally verify our prediction, we have constitutively overexpressed *Ror_a_* in human osteosarcoma cells harbouring a circadian reporter (*Bmal_1_-promoter-luc*) [42], [89] which resulted in loss of oscillations, in agreement with our modelling data (Figure 7B). Higher signals of luciferase activity in *Rora* overexpressing cells than in GFP controls indicate that overexpression was effective since RORa is a known activator of *Bmal1* transcription.

Constitutive overexpression of *Rev-Erbα* dose-dependently dampened circadian oscillation (Figure 7D). Furthermore, higher levels of *Rev-Erbα* decreased *Bmal1-promoter* driven luciferase activity as expected for the transcriptional repressor of Bmal1 [46]. We are aware that a dampening of luminescence signals can reflect both a desynchronization of the cells and a dampening of single cell rhythm. The stronger decay observed after overexpressing both *Ror* and *Rev-Erb* (Figure 7b and Figure 7C) is larger than the GFP control. The same can be observed regarding amplitude and magnitude of the oscillations, indicating an effect of the overexpression on the system independent of the desychronization of cells.

## Discussion

We derived and analysed a model for the mammalian circadian clock of intermediate complexity including the most essential cellular processes such as phosphorylation, complex formation, nuclear translocation and transcriptional regulation. Such mathematical models are helpful to gain a quantitative understanding of the dynamical biological system. They aid experimental design and allow the identification of sensitive nodes in the network or the analysis of perturbation effects on the system. Based on available experimental findings on amplitudes and phases (Dataset S1) and using control theory we determined many parameters in a systematic way. However, not all parameters could be calculated in a unique manner. As an example, the phase shift and the amplitude ratio between mRNA and protein allow to specify two parameters but a third one can be varied freely to fine tune the system (Text S2). Moreover, the detailed kinetics of transcriptional activation and inhibition has not been measured. Consequently, our model is consistent with the available data but cannot be regarded as a precise quantitative model of the core clock. Nevertheless, the model can be exploited to gain insight into fundamental questions: How do transcription and degradation control the period? How do feedback loops interact? Is there a more prominent role for the RBR loop than so far described? By posing such questions, simulating and analysing the biological problem, the model can inspire new experiments to test theoretical predictions.

### Dynamics of *Per* degradation

The effect of perturbing the degradation of clock components on the period represents a difficult open question in circadian biology. Due to the complexity of the system results are difficult to predict. We show theoretically that the effect of mRNA degradation of *Per* on the period is non-monotonic which generates regions of opposite period variation. With a continuous increase of the degradation rate of *Per* mRNA it is possible to obtain first a decrease in the period and subsequently an increase. Our findings regarding a non-monotonic behaviour of *Per* can be related and help to explain apparently contradictory reports regarding perturbations on *Per* and effects on the period.

Indications of opposite change in RNA levels have been found experimentally to induce the same change in the period. Dibner *et al*. [43] reduced overall transcription and observed a period shortening. On the other hand, Chen *et al*. [82] reports that, overexpression of *Per* leads to a period shortening as well. We could simulate both situations with our model (Table 3 in Dataset S2) In these experiments, [43], [82] changes in RNA levels (in both directions) lead to a decrease in the period which resembles the *in silico* dual behaviour regarding *Per*. The reported non-monotonic behaviour might as well explain these seemingly opposite experimental results.

### The role of postranslational modifications

Interestingly, *Cry* seems to be very resilient to perturbations (Figure 6). This could be related to the fact that this gene has two sources of inhibition: PER/CRY and REV-ERB_N_ which together could compensate for the increase in transcription levels. We have tested this hypothesis by removing REV-ERB_N_ inhibition. The new network layout has an effect on *Cry* amplitude but does not lead to loss of oscillations on *Cry*. We therefore speculate that this might be related to the fact that *Cry*, in our system, does not hold posttranslational modifications. These results would be in discrepancy with Ueda *et al*. [90], where overexpression of *Cry* leads to loss of oscillation. However in the Ueda study the exogenous *Cry* was given to the cells introducing additional complexity regarding transcriptional and posttranslational modification. It would be conceivable that CRY protein exhibits posttranslational modifications which would eventually account for the loss of oscillations. On the other hand Fan *et al*. [44] showed that addition of cell-permeable CRY (CP-CRY) does not lead to loss of oscillations and this biological scenario might be closer to our model and therefore related to our predictions. Further experiments and an extension of the mathematical model to incorporate posttranslational modifications of *Cry* would be necessary to answer this question, and will be addressed in future work.

### A two-loop design system for the mammalian circadian clock

The combined activity of the CLOCK/BMAL activator and the P/C inhibitor regulates individual genes with different strengths. Moreover, *Bmal* itself is fine-tuned by REV-ERB and ROR, allowing the generation of oscillations with the appropriate amplitude and phase. A further fine control of the relative phase relations is subsequently achieved by tuning the degradation rates for each element. This fact raises many questions regarding the role of degradation in the individual control of the concentration and peak of expression of the clock genes. The development of a new model for the mammalian circadian clock (Figure 2) and its fitting to state of the art experimental facts (Dataset S1) rouse our awareness to the importance of the RBR loop. The conventional idea of a single driving core loop might not account for the complexity of the circadian clock and might not be sufficient to explain the redundancy mechanisms reported and the robustness of the system. Our results indicate that the RBR loop might have a more prominent role than previously thought. We present theoretical data that propose the RBR loop as being relevant for the generation of oscillations with appropriate amplitude and phases. The RBR loop can act as an independent oscillator even if we disrupt the oscillations of the lower PC loop (Figure 3B). Moreover, we demonstrate experimentally that the overexpression of elements of this loop (*Rev-Erb* and *Ror*) can disrupt oscillations of *Bmal* mRNA. Additionally, the cross-connection between *Rev-Erb* and *Cry* can protect the system from external perturbations of *Cry*, due to the inhibitory action of REV-ERB.

Our work brings new insight into circadian biology, it points to alternative scenarios able to explain experimental findings. It also raises important questions and might motivate further theoretical and experimental work to explore the RBR loop. The medical consequences of such findings should also not be overseen given that the RBR loop involves nuclear receptors which play a crucial role in hormonal processes and metabolism. Their disruption is connected to many diseases, they can be pharmacologically manipulated by agonists or antagonists and therefore represent an important drug target. Moreover, nuclear receptors bind hormones which could make them key players in synchronization and entrainment of clocks. Elements of the RBR loop might represent the missing link between central and peripheral clocks and could be involved in tissue specific circadian regulation.

## Materials and Methods

### Modelling data

The model was designed as a system of 19 ODEs and implemented using Matlab R2010a (Mathworks, Cambridge, UK), with a solver for non-stiff systems (ODE45) which implements a Runge-Kutta method. We have used a relative and absolute tolerance of 10^−9^, with an integration step of 0.01. The system of equations was assembled using Hill-type kinetics and mass action kinetics (Text S1, Figure S1). Most parameters were derived from the literature or analytically determined using LTI theory (Text S2). The remaining parameters were found by fitting the expression profiles of the variables to published phase and amplitude values.

The rainbow plots in Figures 6 and 7 were produced using Xppaut, version 5.85 (http://www.math.pitt.edu/~bard/xpp/xpp.html) and the Xppaut subsystem Auto.

### Experimental data

Lentiviral particles containing hRora, hRev-Erbα or GFP overexpression constructs in a pLenti6 backbone (Invitrogen, Karlsruhe, Germany) were generated as described in published reports [89], [91]. For the high-throughput overexpression analysis of Rora, virus production was performed in 96-well format as described in detail in previous studies [89], [91]. After filtration of the supernatant, U-2 OS cells harbouring a *Bmal1*-luciferase reporter were transduced in the presence of protamine sulfate (8 µg/ml, Sigma-Aldrich, Hamburg, Germany). Next day, medium was substituted by a blasticidine containing medium (positive selection for 3 days; 10 µg/ml, Invitrogen, Karlsruhe, Germany). For viral transduction of hRev-Erbα and GFP in a larger scale, U-2 OS reporter cells were transduced with 250, 500 or 1500 µl lentiviral containing supernatant including 8 µg/ml protamine sulfate. One day after transduction, cells were selected for 7 days with 10 µg/ml blastidicidin and subsequently seeded into 96-well plates. For online bioluminescence monitoring cells were synchronized by 1 µM dexamethasone (Sigma-Aldrich, Hamburg, Germany). Bioluminescence was recorded for 7 days in a stacker-equipped TopCount luminometer with a sampling rate of about 0.5 hours. Two independent measurements for GFP and hRora (each n = 3) were performed. Note that due to technical variations the first peak shows variable amplitudes. Dose-dependent overexpression of hRev-Erbα and GFP was monitored with an n = 4 per dosage. Raw data were de-trended by dividing a 24 h-running average. Periods and amplitudes were estimated by fitting the cosine wave function via the Chronostar analysis software [92]. For visualization, data were smoothened by a 4 hours-running average. Different basal luciferase levels from raw data were included by the fold change in luciferase activity relative to GFP controls for de-trended and smoothed data. Efficiency of dose-dependent hRev-Erbα overexpression was analyzed via quantitative real-time PCR using QuantiTect primer assays (hRev-Erbα QT00000413 and hGAPDH QT01192646; Qiagen, Düsseldorf, Germany).

## Supporting Information

Dataset S1 Collection of mutational and biochemical data for core-clock genes.
(DOC)

Dataset S2 *In silico* results for the period of the system as an effect of perturbing, separately, the degradation, translation and transcription rates.
(DOC)

Figure S1 A model for the mammalian circadian clock. The model comprises two major compartments, the nucleus (light grey) and the cytoplasm. There are 20 species including 5 genes (highlighted in blue boxes), their corresponding cytoplasmic proteins and cytoplasmic protein complexes (indexed “C” and highlighted in violet boxes) and nuclear proteins and nuclear protein complexes (indexed “N” and highlighted in yellow boxes). Dead-end orange lines represent transcription inhibition reactions, brown lines represent complex formation/dissociation reactions and green arrows show other reactions (transcription, translation, import/export and phosphorylation/dephosphoryplation). The dashed horizontal line visually divides the model into two large subunits: the RBR loop and the PC loop. Represented are all parameters and variables used in the construction of the model equations (see Text S1).
(EPS)

Figure S2 *In silico* expression profiles for the RBR loop. The RBR loop is a low amplitude oscillator given a constitutive PC loop (see Figure 3 B). Represented here is the behaviour of the system for different mean values of PC. In a set of 6 *in silico* experiments the PC wild type value (*PC_WT_* = 1.7) is perturbed to: (**A**) +50%; (**B**) +20%; (**C**) +10%; (**D**) −10%; (**E**) −20%; (**F**) −50%. As shown in the figure the oscillations are preserved also under these conditions.
(EPS)

Table S1 Robustness analysis.
(XLS)

Text S1 Model design.
(DOC)

Text S2 Model linearization and parameter determination.
(DOC)
