# Supplementary material for: Tuning the Mammalian Circadian Clock: Robust Synergy of Two Loops
Source: PLoS Comput Biol. 2011 Dec 15;7(12):e1002309. doi: 10.1371/journal.pcbi.1002309 (PMC3240597; doi:10.1371/journal.pcbi.1002309)
Supplement: Dataset S1 — Collection of mutational and biochemical data for core-clock genes. (DOC) [file pcbi.1002309.s001.doc]

**Collection of mutational and biochemical data for core-clock genes.**

| ***Gene*** | **Function** | **Mutation phenotype (mouse) [hours]** | **Peak of RNA expression in the wild type [hours]** | **Peak of Protein expression in the wild type [hours]** |
| --- | --- | --- | --- | --- |
| ***Clock*** | transcription factor | 0.5 shorter period [1] | Constitutive [2] | Constitutive [2] |
| ***ClockΔ19*** | transcription factor | 4 longer period; arrhythmic in DD [2,3,4] | - | - |
| ***Npas*** | transcription factor; Clock paralogue | 0.2 shorter period [2,5,6] | - | - |
| ***Bmal1*** | transcription factor | arrhythmic in DD [2,4] | 15-18 (DD) [2,4,7] | 0-8 [2,4] |
|  |  |  | 16-20 (DD) [5] |  |
|  |  |  | 12-16 (LD);12-20 (DD) [8] |  |
| ***Bmal2*** | transcription factor,  paralogue of Bmal1 | - | - | - |
| ***Per1*** | PER/CRY interaction; CLOCK:BAML1 inhibitor | up to 1.1 shorter period; arrhythmic in DD [2,4] | 4-6 [2] | 10-14 [2] |
|  |  |  | 4 (LD);8 (DD) [8] | 12 (nuclear) [7] |
|  |  |  | 4-8 (DD) [5] |  |
| ***Per2*** | PER/CRY interaction; CLOCK:BAML1 inhibitor | 1.5 shorter period; arrhythmic [2,4] | 6-12 [2] | 10-14 [2] |
|  |  |  | 12 (LD);8 (DD) [8] | 12 (nuclear) [7] |
|  |  |  | 8-12 (DD) [5] |  |
| ***Per3*** | PER/CRY interaction | up to 0.5 shorter period [2,4] | 4-9 [2] | 10 [2] |
| ***Per1+Per3*** | - | short period; arrhythmic [4] | - | - |
| ***Per2+Per3*** | - | short period; arrhythmic [4] |  |  |
| ***Per1+Per2*** | - | Arrhythmic [4] |  |  |
| ***Cry1*** | PER/CRY interaction; CLOCK:BAML1 inhibitor | 1 shorter period [2,4] | 8-12 [2] | 12-18 [2] |
|  |  |  | 12 (LD); 12 (DD) [8] | 12-17 (nuclear) [9] |
|  |  |  | 8-12 (DD) [7] |  |
|  |  |  | 8-12 (LD) [9] |  |
| ***Cry2*** | PER/CRY interaction; CLOCK:BAML1 inhibitor | 1 longer period [2,4] | 8-16 [2] | 12-16 [2] |
|  |  |  | 12-18 (LD) [9] | 12-16 (nuclear) [9] |
| ***Cry1+Cry2*** | - | Arrhythmic [4] | - | - |
| ***Rev-erbα*** | inhibitor of Bmal1 | 0.4 shorter period [2,4] | 2-6 [2] | *8 (in the liver; the RNA, in the liver, has its expression peak at 6 ) [10]* |
|  |  |  | 4 (LD, DD) [11] |  |
|  |  |  | 4 (DD) [8] |  |
|  |  |  | 2-6 (DD) [5] |  |
| ***Rev-erbβ*** | inhibitor of Bmal1 | - | - | - |
| ***Rora*** | activator of Bmal1 | 0.5 shorter period (*staggerer* mutant) [12] | 8 (DD) [8] | - |
| ***Rorb*** | activator of Bmal1 | 0.5 longer period [13] | - | - |
| ***Rorc*** | activator of Bmal1 | - | - | - |
| ***CK1*** | phosphorylation of PERs, CRYs, and BMAL1 | 4 shorter period (*tau* mutant) [2,4] | - | - |

**Table 1.** Components of the mammalian circadian clock. Mutational phenotypes concern the mouse model organism. All measurements regarding peaks of expression are retrieved from SCN tissue (data marked in italic is extracted from liver tissue).

| ***Gene*** | **Peak of RNA expression in the wild type**  **[hours]** | **Peak of Protein expression in the wild type [hours]** | **Normalized amplitudes of RNA expression in the wild type** |
| --- | --- | --- | --- |
| ***Bmal1*** | 15-18(DD) [2,4,7] | 0-8 [2,4] | 0.25 DD [5] |
|  | 16-20(DD) [5] |  | 0.3 LD [8] |
|  | 12-16(LD);12-20(DD) [8] |  | 0.26 DD [8] |
| ***Per2*** | 6-12 [2] | 10-14 [2] | 0.36 LD [14] |
|  | 12 (LD);8(DD) [8] | 12 (nuclear) [7] | 0.71 DD [8] |
|  | 8-12 (DD) [5] |  | 0.39 DD [5] |
| ***Cry1*** | 8-12 [2] | 12-18 [2] | 0.12 LD [9] |
|  | 12(LD); 12(DD) [8] | 12-17 (nuclear) [9] | 0.33 LD [8] |
|  | 8-12 (DD) [7] |  | 0.2 DD [8] |
|  | 8-12(LD) [9] |  |  |
| ***Rev-erbα*** | 2-6 [2] | *8 (in the liver; the RNA, in the liver, has its expression peak at 6 ) [10]* | 0.42 DD [5] |
|  | 4(LD&DD) [11] |  | 0.6 DD [11] |
|  | 4(DD) [8] |  | 0.67 LD [11] |
|  | 2-6(DD) [5] |  | 0.54 DD [8] |
| ***Rora*** | 8(DD) [8] | - | 0.43 DD [8] |

**Table 2.** Circadian times and normalized amplitudes for core clock genes. All measurements are retrieved from SCN tissue. DD, dark-dark conditions; LD, light-dark conditions.

| ***Gene*** | **m RNA half-life**  **[hours]** | **Cell lines/tissues** | **protein half-life**  **[hours]** | **Cell lines/tissues** |
| --- | --- | --- | --- | --- |
| ***Bmal1*** | 2.39 [15]  1.66-0.67 [16] | Mouse fibroblasts |  |  |
| ***Clock*** | 2.64 [15] | Mouse fibroblasts |  |  |
|  | 4.06 [17] | Mouse embryonic stem cells |  |  |
| ***Per1*** | 2.45 [15] | Human B cells |  |  |
|  | 3.72 [17] | Mouse embryonic stem cells |  |  |
| ***Per2*** | 0.87 [15] | Mouse fibroblasts | 2.5 [18] | COS7 cells |
|  | 3.04 [15] | Human B cells | 2.5-3 [19] | NIH3T3 cells |
|  | 2.88 [17] | Mouse embryonic stem cells |  |  |
| ***Per3*** | 3.64 [15] | Mouse fibroblasts |  |  |
|  | 5.92 [15] | Human B cells |  |  |
| ***Cry1*** | 3.86 [15] | Mouse fibroblasts | 3 [20] | COS7 cells |
|  | 4.35 [17] | Mouse embryonic stem cells |  |  |
| ***Cry2*** | 4.2 [17] | Mouse embryonic stem cells | 2.5 [20] | COS7 cells |
| ***Rev-erbα*** | 3.66 [17] | Mouse embryonic stem cells | 2 [21] | Hela cells |
| ***Rev-erbβ*** | 3.02 [15] | Mouse fibroblasts |  |  |
|  | 5.50 [15] | Human B cells |  |  |
|  | 3.61 [17] | Mouse embryonic stem cells |  |  |
| ***Rora*** | 3.14 [17] | Mouse embryonic stem cells | 1.3 [22] | COS1 cells |
| ***Rorb*** | 5.68 [17] | Mouse embryonic stem cells |  |  |
| ***Rorc*** | 4.97 [17] | Mouse embryonic stem cells |  |  |

**Table 3**. Half-life times of core clock components, mRNA and proteins.

References

1. Debruyne JP, Noton E, Lambert CM, Maywood ES, Weaver DR, et al. (2006) A clock shock: mouse CLOCK is not required for circadian oscillator function. Neuron 50: 465-477.

2. Lowrey PL, Takahashi JS (2004) Mammalian circadian biology: elucidating genome-wide levels of temporal organization. Annu Rev Genomics Hum Genet 5: 407-441.

3. Antoch MP, Song EJ, Chang AM, Vitaterna MH, Zhao Y, et al. (1997) Functional identification of the mouse circadian Clock gene by transgenic BAC rescue. Cell 89: 655-667.

4. Reppert SM, Weaver DR (2002) Coordination of circadian timing in mammals. Nature 418: 935-941.

5. DeBruyne JP, Weaver DR, Reppert SM (2007) CLOCK and NPAS2 have overlapping roles in the suprachiasmatic circadian clock. Nat Neurosci 10: 543-545.

6. Dudley CA, Erbel-Sieler C, Estill SJ, Reick M, Franken P, et al. (2003) Altered patterns of sleep and behavioral adaptability in NPAS2-deficient mice. Science 301: 379-383.

7. Shearman LP, Sriram S, Weaver DR, Maywood ES, Chaves I, et al. (2000) Interacting molecular loops in the mammalian circadian clock. Science 288: 1013-1019.

8. Ueda HR, Chen W, Adachi A, Wakamatsu H, Hayashi S, et al. (2002) A transcription factor response element for gene expression during circadian night. Nature 418: 534-539.

9. Kume K, Zylka MJ, Sriram S, Shearman LP, Weaver DR, et al. (1999) mCRY1 and mCRY2 are essential components of the negative limb of the circadian clock feedback loop. Cell 98: 193-205.

10. Preitner N, Damiola F, Lopez-Molina L, Zakany J, Duboule D, et al. (2002) The orphan nuclear receptor REV-ERBalpha controls circadian transcription within the positive limb of the mammalian circadian oscillator. Cell 110: 251-260.

11. Onishi H, Yamaguchi S, Yagita K, Ishida Y, Dong X, et al. (2002) Rev-erbalpha gene expression in the mouse brain with special emphasis on its circadian profiles in the suprachiasmatic nucleus. J Neurosci Res 68: 551-557.

12. Sato TK, Panda S, Miraglia LJ, Reyes TM, Rudic RD, et al. (2004) A functional genomics strategy reveals Rora as a component of the mammalian circadian clock. Neuron 43: 527-537.

13. Andre E, Conquet F, Steinmayr M, Stratton SC, Porciatti V, et al. (1998) Disruption of retinoid-related orphan receptor beta changes circadian behavior, causes retinal degeneration and leads to vacillans phenotype in mice. EMBO J 17: 3867-3877.

14. Jin X, Shearman LP, Weaver DR, Zylka MJ, de Vries GJ, et al. (1999) A molecular mechanism regulating rhythmic output from the suprachiasmatic circadian clock. Cell 96: 57-68.

15. Friedel CC, Dolken L, Ruzsics Z, Koszinowski UH, Zimmer R (2009) Conserved principles of mammalian transcriptional regulation revealed by RNA half-life. Nucleic Acids Res 37: e115.

16. Schibler U, Suter DM, Molina N, Gatfield D, Schneider K, et al. (2011) Mammalian Genes Are Transcribed with Widely Different Bursting Kinetics. Science 332: 472-474.

17. Sharova LV, Sharov AA, Nedorezov T, Piao Y, Shaik N, et al. (2009) Database for mRNA half-life of 19 977 genes obtained by DNA microarray analysis of pluripotent and differentiating mouse embryonic stem cells. DNA Res 16: 45-58.

18. Meng QJ, Logunova L, Maywood ES, Gallego M, Lebiecki J, et al. (2008) Setting clock speed in mammals: the CK1 epsilon tau mutation in mice accelerates circadian pacemakers by selectively destabilizing PERIOD proteins. Neuron 58: 78-88.

19. Vanselow K, Vanselow JT, Westermark PO, Reischl S, Maier B, et al. (2006) Differential effects of PER2 phosphorylation: molecular basis for the human familial advanced sleep phase syndrome (FASPS). Genes Dev 20: 2660-2672.

20. Godinho SI, Maywood ES, Shaw L, Tucci V, Barnard AR, et al. (2007) The after-hours mutant reveals a role for Fbxl3 in determining mammalian circadian period. Science 316: 897-900.

21. Yin L, Wang J, Klein PS, Lazar MA (2006) Nuclear receptor Rev-erbalpha is a critical lithium-sensitive component of the circadian clock. Science 311: 1002-1005.

22. Moraitis AN, Giguere V (2003) The co-repressor hairless protects RORalpha orphan nuclear receptor from proteasome-mediated degradation. J Biol Chem 278: 52511-52518.
