# Supplementary material for: Tuning the Mammalian Circadian Clock: Robust Synergy of Two Loops
Source: PLoS Comput Biol. 2011 Dec 15;7(12):e1002309. doi: 10.1371/journal.pcbi.1002309 (PMC3240597; doi:10.1371/journal.pcbi.1002309)
Supplement: Dataset S2 — In silico results for the period of the system as an effect of perturbing, separately, the degradation, translation and transcription rates. (DOC) [file pcbi.1002309.s002.doc]

***In silico* results for the period of the system as an effect of perturbing, separately, the degradation, translation and transcription rates.**

| **mRNA** | **degradation rate** | **WT** | **+10%** | **T** | **ΔT %** | **+50%** | **T** | **ΔT %** | **+70%** | **T** | **ΔT %** | **2 fold increase** | **T** | **ΔT %** | ***T for 2 fold increase (transients)*** | **Δ*T %*** |
| --- | --- | --- | --- | --- | --- | --- | --- | --- | --- | --- | --- | --- | --- | --- | --- | --- |
| ***Per*** | $d_{y1}$ | 0.3 | 0.33 | 23.6 | **WT** | 0.45 | 23.7 | **1** | 0.51 | 24.5 | **4.1** | 0.6 | 24.1 | **2.6** | *23.8* | ***1.3*** |
| ***Cry*** | $d_{y2}$ | 0.2 | 0.22 | 23.6 | **WT** | 0.3 | 23.6 | **WT** | 0.34 | 23.9 | **1.8** | 0.4 | 23.6 | **WT** | *23.6* | ***WT*** |
| ***Rev-Erb*** | $d_{y3}$ | 2 | 2.2 | 23.4 | **WT** | 3 | AR |  | 3.4 | AR |  | 4 | AR |  | *19.6* | ***-20.1 (AR)*** |
| ***Ror*** | $d_{y4}$ | 0.2 | 0.22 | 23 | **-2.1** | 0.3 | AR |  | 0.34 | AR |  | 0.4 | AR |  | *22.1* | ***-6.3 (AR)*** |
| ***Bmal*** | $d_{y5}$ | 1.6 | 1.76 | 23.5 | **WT** | 2.4 | AR |  | 2.72 | AR |  | 3.2 | AR |  | *22.8* | ***-3***  ***(AR)*** |

**Table 1.** Perturbation of the degradation rates has an effect on the period. We varied the RNA degradation rates from 10% to a 2 fold increase to the wild type (T_WT_ = 23.5) value and measured the period of *Bmal* as an outcome of the simulations. The new values for the degradation rates are indicated (+10%, +50%, +70% and 2 fold increase), as well as, the new value for the period (T) and the corresponding variation to the wild type value (ΔT %). Positive values indicate an increase in the period, negative values a decrease and *WT* indicates variations between +/- 1% to the wild type value. The last two columns of the table concern the transient region of the simulations. Interestingly, for an increase of the RNA degradation rate in members of the lower PC loop there is an increase or no change in the period. If the same perturbation is applied to members of the upper RBR loop a decrease in the period can be measured followed by loss of oscillations (AR).

| **protein** | **translation rate** | **WT** | **-10%** | **T** | **Δ T %** | **-50%** | **T** | **ΔT %** | **-70%** | **T** | **ΔT %** | ***T for 2 fold decrease (transients)*** | **Δ*T %*** |
| --- | --- | --- | --- | --- | --- | --- | --- | --- | --- | --- | --- | --- | --- |
| **PER_C_** | $k_{p1}$ | 0.4 | 0.36 | 23.8 | **1.2** | 0.2 | 24.3 | **3.3** | 0.12 | 24.7 | 5 | *24.7* | ***4.7*** |
| **CRY_C_** | $k_{p2}$ | 0.26 | 0.23 | 23.6 | **WT** | 0.13 | 24.2 | **2.7** | 0.08 | 24.7 | 4.8 | *24.4* | ***3.6*** |
| **REV-ERB_C_** | $k_{p3}$ | 0.37 | 0.33 | 23.6 | **WT** | 0.19 | AR |  | 0.11 | AR |  | *19.1* | ***-22.9 (AR)*** |
| **ROR_C_** | $k_{p4}$ | 0.76 | 0.68 | 23.3 | **WT** | 0.38 | AR |  | 0.23 | AR |  | *22.5* | ***-4.6 (AR)*** |
| **BMAL_C_** | $k_{p5}$ | 1.21 | 1.09 | 23.3 | **WT** | 0.61 | AR |  | 0.36 | AR |  | *23* | ***-2 (AR)*** |

**Table 2.** Variation of the translation rates has an effect on the period. We varied the translation rates rates from 10% to a 2 fold decrease to the wild type (WT) value and measured the period of *Bmal* as an outcome of the simulations. The new values for the translation rates are indicated (-10%, -50% and -70%), as well as, the new value for the period (T) and the corresponding variation to the wild type value (ΔT %). Positive values indicate an increase in the period, negative values a decrease and *WT* indicates variations between +/- 1% to the wild type value. The last two columns of the table concern the transient region of the simulations. As remarked in Table 1, for a decrease of the translation rate in members of the lower PC loop there is an increase or no change in the period, on the contrary, if the same perturbation is applied to members of the upper RBR loop a decrease in the period can be measured followed by loss of oscillations (AR). This data is in agreement with the data obtained as a result of perturbing the degradation rate (Table 1).

| **mRNA** | **transcription rate** | **WT** | **10 fold decrease** | **T** | **Δ*T %*** | **-50 %** | **T** | **Δ*T %*** | **-10 %** | **T** | **Δ*T %*** | **+10 %** | **T** | **Δ*T %*** | **+50 %** | **T** | **Δ*T %*** | **10 fold increase** | **T** | **Δ*T %*** |
| --- | --- | --- | --- | --- | --- | --- | --- | --- | --- | --- | --- | --- | --- | --- | --- | --- | --- | --- | --- | --- |
| ***Per*** | $V_{1\max}$ | 1 | 0.1 | AR |  | 0.5 | 24.3 | ***3.4*** | 0.9 | 23.9 | ***1.5*** | 1.1 | 23.6 | ***WT*** | 1.5 | 22.9 | ***-2.8*** | 10 | AR |  |
| ***Cry*** | $V_{2\max}$ | 2.92 | 0.29 | 25.3 | ***7*** | 1.46 | 24.2 | ***2.7*** | 2.63 | 23.6 | ***WT*** | 3.21 | 23.6 | ***WT*** | 4.38 | 23.6 | ***WT*** | 29 | 23.4 | ***WT*** |
| ***Rev-Erb*** | $V_{3\max}$ | 1.9 | 0.19 | AR |  | 0.95 | AR |  | 1.71 | 23.8 | ***1.1*** | 2.09 | 23.6 | ***WT*** | 2.85 | AR |  | 19 | AR |  |
| ***Ror*** | $V_{4\max}$ | 10.9 | 1.09 | AR |  | 5.45 | AR |  | 9.81 | 23.2 | ***-1.1*** | 11.99 | 23.8 | ***1.3*** | 16.35 | 24.1 | ***2.6*** | 109 | AR |  |
| ***Bmal*** | $V_{5\max}$ | 1 | 0.1 | AR |  | 0.5 | AR |  | 0.9 | 23.4 | ***WT*** | 1.1 | 24.8 | ***5.2*** | 1.5 | 24.5 | ***4*** | 10 | AR |  |
| ***All*** | | |  | ***AR*** |  |  | ***AR*** |  |  | ***23.4*** | ***WT*** |  | ***24*** | ***1.9*** |  | *24.2* | ***2.8*** |  | *47.2* | ***50.2 (AR)*** |

**Table 3.** Variation of the transcription rates has an effect on the period. We varied the translation rates from a 10 fold decrease to a 10 fold increase to the wild type (WT) value and measured the period of *Bmal* as an outcome of the simulations. The new values for the transcription rates are indicated (10 fold increase/decrease; +/- 50% and +/- 10%), as well as, the new value for the period (T) and the corresponding variation to the wild type value (ΔT %). Positive values indicate an increase in the period and negative values a decrease, *WT* indicates variations between +/- 1% to the wild type value. The last row shows the effect on the system when applying a perturbation to all 5 genes simultaneously.

| **Mutant simulated** | **Parameter perturbed** | **WT** | **mutant** | **T** | **Δ*T %*** |
| --- | --- | --- | --- | --- | --- |
| **CLOCK** | $kf_{x1}$ | 2.3 | 0.5 | 25.6 | **8.4** |
| **CK1ε** | $kf_{z4}$ | 1 | 0.1 | 24.3 | **3.4** |
|  | $kf_{z5}$ | 1 | 0.1 |  |  |

**Table 4.** The model generated is able to simulate CLOCK and CK1ε mutational phenotypes. Represented are the parameters perturbed to simulate each mutant as well as the wild type and the perturbed value. The period of the system was measured on *Bmal* expression profile, before and after perturbation. The new value for the period (T) and the corresponding variation to the wild type value is indicated (**Δ**T %).
