# Supplementary material for: Tuning the Mammalian Circadian Clock: Robust Synergy of Two Loops
Source: PLoS Comput Biol. 2011 Dec 15;7(12):e1002309. doi: 10.1371/journal.pcbi.1002309 (PMC3240597; doi:10.1371/journal.pcbi.1002309)
Supplement: Text S1 — Model design. (DOC) [file pcbi.1002309.s006.doc]

**Model design**

The model presented for the mammalian circadian clock was designed based on the information contained on Figure S1. This figure contains the same elements as Figure 1 in the main text and additionally all parameters and variables indicated on the equations. The model comprises two major compartments, the nucleus (light grey) and the cytoplasm. There are 20 species represented, including, 5 genes (highlighted in blue boxes); their corresponding cytoplasmic proteins and cytoplasmic protein complexes (indexed “C” and highlighted in violet boxes) and nuclear proteins and nuclear protein complexes (indexed “N” and highlighted in yellow boxes). Dead-end orange lines represent transcription inhibition reactions, green arrows other reactions (transcription, translation, import/export, phosphorylation/dephosphoryplation, and complex formation). The dashed horizontal line visually divides the model into two large subunits: the RBR loop and the PC loop. All parameters and variables are given in, Table 2A, 2B and 2C. All time units are given in hours and concentration units are given as arbitrary units (a.u.). It should be noted that arbitrary units cannot be compared across different publications according to IUPAC rules [1].

The network in Figure S1 can be translated into the following set of ordinary differential equations:

CLOCK/BMAL

$\frac{dx1}{dt}=kf_{x1}x7-kd_{x1}x1-d_{x1}x1$ (1)

*Rev-Erb*

$\frac{dy3}{dt}=V_{3\max}\frac{1+g\left(\frac{x1}{k_{t3}}\right)^{v}}{1+\left(\frac{PC}{k_{i3}}\right)^{w}\left(\frac{x1}{k_{t3}}\right)^{v}+\left(\frac{x1}{k_{t3}}\right)^{v}}-d_{y3}y3$ (2)

*Ror*

$\frac{dy4}{dt}=V_{4\max}\frac{1+h\left(\frac{x1}{k_{t4}}\right)^{p}}{1+\left(\frac{PC}{k_{i4}}\right)^{q}\left(\frac{x1}{k_{t4}}\right)^{p}+\left(\frac{x1}{k_{t4}}\right)^{p}}-d_{y4}y4$ (3)

REV-ERB_C_

$\frac{dz6}{dt}=k_{p3}(y3+y3_{0})-ki_{z6}z6-d_{z6}z6$ (4)

ROR_C_

$\frac{dz7}{dt}=k_{p4}(y4+y4_{0})-ki_{z7}z7-d_{z7}z7$ (5)

REV-ERB_N_

$\frac{dx5}{dt}=ki_{z6}z6-d_{x5}x5$ (6)

ROR_N_

$\frac{dx6}{dt}=ki_{z7}z7-d_{x6}x6$ (7)

*Bmal*

$\frac{dy5}{dt}=V_{5\max}\frac{1+i\left(\frac{x6}{k_{t5}}\right)^{n}}{1+\left(\frac{x5}{k_{i5}}\right)^{m}+\left(\frac{x6}{k_{t5}}\right)^{n}}-d_{y5}y5$ (8)

BMAL_C_

$\frac{dz8}{dt}=k_{p5}(y5+y5_{0})-ki_{z8}z8-d_{z8}z8$ (9)

BMAL_N_

$\frac{dx7}{dt}=ki_{z8}z8+kd_{x1}x1-kf_{x1}x7-d_{x7}x7$ (10)

*Per*

$\frac{dy1}{dt}=V_{1\max}\frac{1+a\left(\frac{x1}{k_{t1}}\right)^{b}}{1+\left(\frac{PC}{k_{i1}}\right)^{c}\left(\frac{x1}{k_{t1}}\right)^{b}+\left(\frac{x1}{k_{t1}}\right)^{b}}-d_{y1}y1$ (11)

*Cry*

$\frac{dy2}{dt}=V_{2\max}\frac{1+d\left(\frac{x1}{k_{t2}}\right)^{e}}{1+\left(\frac{PC}{k_{i2}}\right)^{f}\left(\frac{x1}{k_{t2}}\right)^{e}+\left(\frac{x1}{k_{t2}}\right)^{e}}\frac{1}{1+\left(\frac{x5}{k_{i21}}\right)^{f1}}-d_{y2}y2$ (12)

CRY_C_

$\frac{dz1}{dt}=k_{p2}(y2+y2_{0})+kd_{z4}z4+kd_{z5}z5-kf_{z5}z1z2-kf_{z4}z1z3-d_{z1}z1$ (13)

PER_C_

$\frac{dz2}{dt}=k_{p1}(y1+y1_{0})+kd_{z5}z5+kd_{phz3}z3-kf_{z5}z2z1-kph_{z2}z2-d_{z2}z2$ (14)

PER_C_^*^

$\frac{dz3}{dt}=kph_{z2}z2+kd_{z4}z4-kd_{phz3}z3-kf_{z4}z3z1-d_{z3}z3$ (15)

PER_C_^*^/CRY_C_

$\frac{dz4}{dt}=kf_{z4}z1z3+ke_{x2}x2-ki_{z4}z4-kd_{z4}z4-d_{z4}z4$ (16)

PER_C_/CRY_C_

$\frac{dz5}{dt}=kf_{z5}z1z2+ke_{x3}x3-ki_{z5}z5-kd_{z5}z5-d_{z5}z5$ (17)

PER^*^_N_/CRY_N_

$\frac{dx2}{dt}=ki_{z4}z4-ke_{x2}x2-d_{x2}x2$ (18)

PER_N_/CRY_N_

$\frac{dx3}{dt}=ki_{z5}z5-ke_{x3}x3-d_{x3}x3$ (19)

PER/CRY_pool_

$PC=x2+x3$ (20)

List of Variables

| **Variable [a.u.]** | **Name** |
| --- | --- |
| ***x1*** | CLOCK/BMAL |
| ***x2*** | PER^*^_N_/CRY_N_ |
| ***x3*** | PER_N_/CRY_N_ |
| ***PC*** | PER/CRY_pool_ |
| ***x5*** | REV-ERB_N_ |
| ***x6*** | ROR_N_ |
| ***y1*** | *Per* |
| ***y2*** | *Cry* |
| ***y3*** | *Rev-Erb* |
| ***y4*** | *Ror* |
| ***y5*** | *Bmal* |
| ***z1*** | CRY_C_ |
| ***z2*** | PER_C_ |
| ***z3*** | PER_C_^*^ |
| ***z4*** | PER_C_^*^/CRY_C_ |
| ***z5*** | PER_C_/CRY_C_ |
| ***z6*** | REV-ERB_C_ |
| ***z7*** | ROR_C_ |
| ***z8*** | BMAL_C_ |

**Table 1.** List of Variables

List of Parameters

| **Parameters** | **Name** | **Value** | | |
| --- | --- | --- | --- | --- |
| **Degradation rates for nuclear proteins or nuclear protein complexes [hour^-1^]** | | | | |
| ***d_x1_*** | CLOCK/BMAL | | | 0.08 |
| ***d_x2_*** | PER^*^_N_/CRY_N_ | | | 0.06 |
| ***d_x3_*** | PER_N_/CRY_N_ | | | 0.09 |
| ***d_x5_*** | REV-ERB_N_ | | | 0.17 |
| ***d_x6_*** | ROR_N_ | | | 0.12 |
| ***d_x7_*** | BMAL_N_ | | | 0.15 |
| **Degradation rates for mRNAs [hour^-1^]** | | | | |
| ***d_y1_*** | *Per* | | | 0.3 |
| ***d_y2_*** | *Cry* | | | 0.2 |
| ***d_y3_*** | *Rev-Erb* | | | 2 |
| ***d_y4_*** | *Ror* | | | 0.2 |
| ***d_y5_*** | *Bmal* | | | 1.6 |
| **Degradation rates for cytoplasmic proteins [hour^-1^]** | | | | |
| ***d_z1_*** | CRY_C_ | | | 0.23 |
| ***d_z2_*** | PER_C_ | | | 0.25 |
| ***d_z3_*** | PER_C_^*^ | | | 0.6 |
| ***d_z4_*** | PER_C_^*^/CRY_C_ | | | 0.2 |
| ***d_z5_*** | PER_C_/CRY_C_ | | | 0.2 |
| ***d_z6_*** | REV-ERB_C_ | | | 0.31 |
| ***d_z7_*** | ROR_C_ | | | 0.3 |
| ***d_z8_*** | BMAL_C_ | | | 0.73 |
| **Reaction rates for complex formation/dissociation** | | | | |
| ***kf_x1_*** | CLOCK/BMAL-complex formation **[hour^-1^]** | | | 2.3 |
| ***kd_x1_*** | CLOCK/BMAL-complex dissociation **[hour^-1^]** | | | 0.01 |
| ***kf_z4_*** | PER_C_^*^/CRY_C_-complex formation **[(a.u.hour)^-1^]** | | | 1 |
| ***kd_z4_*** | PER_C_^*^/CRY_C_-complex dissociation **[hour^-1^]** | | | 1 |
| ***kf_z5_*** | PER_C_/CRY_C_-complex formation **[(a.u.hour)^-1^]** | | | 1 |
| ***kd_z5_*** | PER_C_/CRY_C_-complex dissociation **[hour^-1^]** | | | 1 |
| **Phosphorylation/dephosphorylation reaction rates [hour^-1^]** | | | | |
| ***kph_z2_*** | PER_C-_phosphorylation rate | | 2 | |
| ***kd_phz3_*** | PER_C_^*-^dephosphorylation rate | | 0.05 | |
| **Transcription rates [a.u.hour^-1^]** | | | | |
| ***V_1max_*** | *Per* | | | 1 |
| ***V_2max_*** | *Cry* | | | 2.92 |
| ***V_3max_*** | *Rev-Erb* | | | 1.9 |
| ***V_4max_*** | *Ror* | | | 10.9 |
| ***V_5max_*** | *Bmal* | | | 1 |

**Table 2A.** List of Parameters

| **Parameters** | | **Name** | **Value** | |
| --- | --- | --- | --- | --- |
| **Activation/inhibition rates [a.u.]** | | | | |
| ***k_t1_*** | *Per*-activation rate | | | 3 |
| ***k_i1_*** | *Per*-inhibition rate | | | 0.9 |
| ***k_t2_*** | *Cry*-activation rate | | | 2.4 |
| ***k_i2_*** | *Cry*-inhibition rate | | | 0.7 |
| ***k_i21_*** | *Cry*-inhibition rate | | | 5.2 |
| ***k_t3_*** | *Rev-Erb*-activation rate | | | 2.07 |
| ***k_i3_*** | *Rev-Erb*-inhibition rate | | | 3.3 |
| ***k_t4_*** | *Ror*-activation rate | | | 0.9 |
| ***k_i4_*** | *Ror*-inhibition rate | | | 0.4 |
| ***k_t5_*** | *Bmal*-activation rate | | | 8.35 |
| ***k_i5_*** | *Bmal*-inhibition rate | | | 1.94 |
| **Transcription fold activation (dimensionless)** | | | | |
| ***a*** | *Per* | | | 12 |
| ***d*** | *Cry* | | | 12 |
| ***g*** | *Rev-Erb* | | | 5 |
| ***h*** | *Ror* | | | 5 |
| ***i*** | *Bmal* | | | 12 |
|  | **Production rates [hour^-1^]** | | |  |
| ***k_p1_*** | PER_C_ | | | 0.4 |
| ***k_p2_*** | CRY_C_ | | | 0.26 |
| ***k_p3_*** | REV-ERB_C_ | | | 0.37 |
| ***k_p4_*** | ROR_C_ | | | 0.76 |
| ***k_p5_*** | BMAL_C_ | | | 1.21 |
|  | **Import/Export rates [hour^-1^]** | | |  |
| ***ki_z4_*** | PER_C_^*^/CRY_C_ | | | 0.2 |
| ***ki_z5_*** | PER_C_/CRY_C_ | | | 0.1 |
| ***ki_z6_*** | REV-ERB_C_ | | | 0.5 |
| ***ki_z7_*** | ROR_C_ | | | 0.1 |
| ***ki_z8_*** | BMAL_C_ | | | 0.1 |
| ***ke_x2_*** | PER^*^_N_/CRY_N_ | | | 0.02 |
| ***ke_x3_*** | PER_N_/CRY_N_ | | | 0.02 |

**Table 2B.** List of Parameters

| **Parameters** | **Name** | **Value** |
| --- | --- | --- |
| **Hill coefficients of transcription (dimensionless)** | | |
| ***b*** | *Per*-activation | 5 |
| ***c*** | *Per*-inhibition | 7 |
| ***e*** | *Cry*-activation rate | 6 |
| ***f*** | *Cry*-inhibition | 4 |
| ***f1*** | *Cry*-inhibition | 1 |
| ***v*** | *Rev-Erb*-activation | 6 |
| ***w*** | *Rev-Erb*-inhibition | 2 |
| ***p*** | *Ror*-activation | 6 |
| ***q*** | *Ror*-inhibition | 3 |
| ***n*** | *Bmal*-activation | 2 |
| ***m*** | *Bmal*-inhibition | 5 |
|  | **Exogenous RNA [a.u.]** |  |
| ***y1_0_*** | *Per* | 0 |
| ***y2_0_*** | *Cry* | 0 |
| ***y3_0_*** | *Rev-Erb* | 0 |
| ***y4_0_*** | *Ror* | 0 |
| ***y5_0_*** | *Bmal* | 0 |

**Table 2C.** List of Parameters

References

1. Olesen H (1995) Properties and units in the clinical laboratory sciences. I. Syntax and semantic rules (recommendation 1995). International Union of Pure and Applied Chemistry (IUPAC) and International Federation of Clinical Chemistry (IFCC). Eur J Clin Chem Clin Biochem 33: 627-636.
