# Supplementary material for: Tuning the Mammalian Circadian Clock: Robust Synergy of Two Loops
Source: PLoS Comput Biol. 2011 Dec 15;7(12):e1002309. doi: 10.1371/journal.pcbi.1002309 (PMC3240597; doi:10.1371/journal.pcbi.1002309)
Supplement: Text S2 — Model linearization and parameter determination. (DOC) [file pcbi.1002309.s007.doc]

**Model linearization and parameter determination.**

We aimed to model the network presented in Figure 1A and to determine parameters analytically from known phase relationships and amplitude ratios of clock components.

**Figure 1.** Analysis of the circadian network by linearization of individual branches. **(A)** represents the full model and **(B)** one biological process.


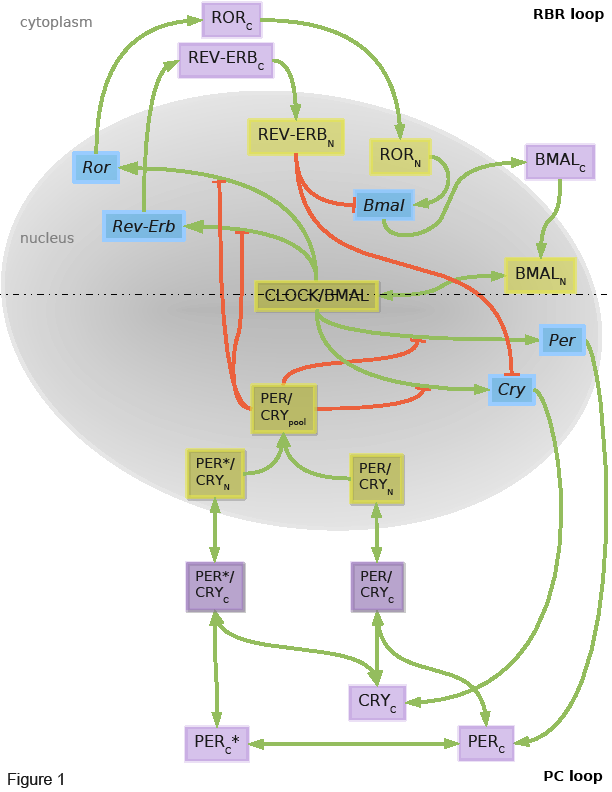

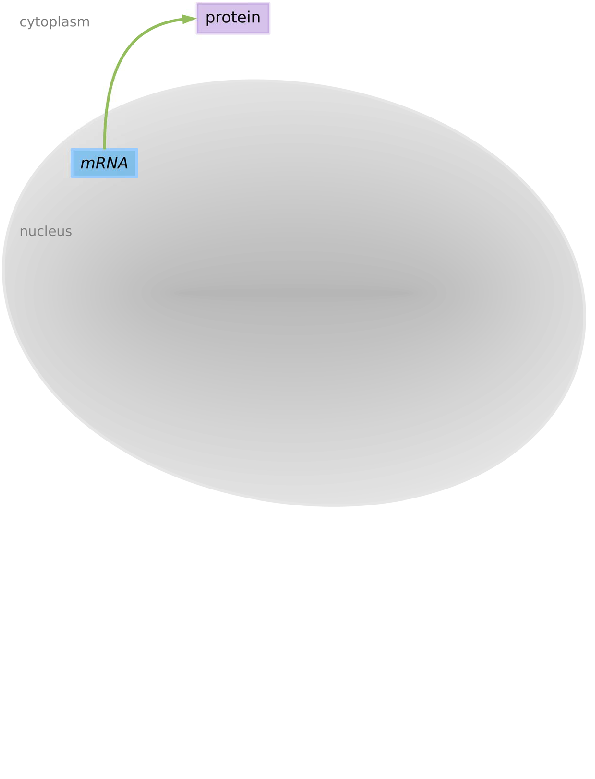


A

B

The choice of reasonable kinetics and the estimation of parameters is a sensitive issue – essentially in every modelling endeavour. Oscillations due to delayed negative feedback loops can be fitted to simple models (single delay-differential equations [1] or Goodwin models with a single nonlinearity) or alternatively one might construct larger more detailed models describing all major clock components including posttranslational modifications, complexes formations etc. Here we have decided to develop a model of *intermediate complexity* including the most essential cellular processes. However, due to the fact that some critical processes (transcriptional regulation, enzymatic degradation) have still not been measured experimentally in sufficient quantitative detail, any model – including ours - has to make some assumption regarding Hill functions and degradation kinetics.

Furthermore, we invested considerable effort to collect available experimental data on phase relations, amplitudes and degradation rates (see Dataset S1, Tables 2 and 3). Our LTI approach provides a direct link from measured data to model parameters.

The use of linear-time-invariant (LTI) systems theory required the simplifcation of the network and the linearization of the correspondent equations [2]. As an illustration of the methodology we present here the procedure regarding a short branch from an mRNA to the corresponding protein (Figure 1B).

The reaction depicted in figure S3B can be described as:

, (1)

*x* represents the mRNA, *k* the translation of the protein, *y* the protein, *t* the export rate and *d* the degradation rate.

This corresponds to the following linear differential equation:

$\frac{dy}{dt}=kx-dy-ty=kx-(d+t)y$ (2)

Using the transfer function we can represent (1) as:

$x\xrightarrow{}G\xrightarrow{}y$ (3)

G is the transfer function which can be determined applying a Fourier transform,

$G=\frac{k}{d+iw}$, (4)

$\left|G\right|=k\frac{1}{\sqrt{(d+t)^{2}+w^{2}}}$ with $w=\frac{2\pi}{T}$, (5)

where T is the period of the system.

If we consider a periodic input, $x=A\sin(wt)$ (6)

then $y=\left|G(iw)\right|A\sin(wt+\phi)$ (7)

And therefore $\phi=\arctan\left(\frac{w}{d+t}\right)$ (8)

The value for *d+t* can be calculated so that *  *the phase diference between *x* and *y* equals* ϕ,*

$\phi=\phi_{y}-\phi_{x}$, (9)

And the value for k can be determined using the relation below,

$\left|G\right|=\frac{yamplitude}{xamplitude}$ (10)

In summary, we started the procedure with three unknown parameters: *k*, *d* and *t* and using the relations (9) and (10) we were able to calculate two of them, so that a third one can be chosen freely. Additional experiments would be necessary to distinguish degradation and nuclear import. Our application of the LTI together with the use of known parameters retrieved from the literature (Dataset S1) can provide 61 of the model parameters. As shown above, lumping together degradation and transport leaves one kinetic parameter undefined. Furthermore, some amplitudes and phases of cytoplasmic and nuclear proteins are not available separately (Dataset S1). Thus 10 parameters have been optimized by fine tuning of phases and amplitudes of the non-linear system.

Finally, our model is consistent with available data. Nevertheless, we are aware and concede that our model (as any other available mammalian clock model) is neither unique nor perfect in all quantitative details. Many parameters represent a kind of effective kinetics, since we lump together genes such as *Per1*, *Per2* and *Per3* and condense complex combinatorial and epigenetic regulations into effective Hill functions. We are convinced, however, that our model is quantitative enough to provide conceptual insight on the effects of transcription and degradation rates and on the role of the feedback loops.

**Detailed description of the model and of parameter determination**

We linearize the model around the steady state and therefore any kinetics can be approximated by linear terms. Linear models have no limit cycles. Thus we consider the feedbacks as open loops forced by a 24 hour oscillation. In this way the parameters were optimized to reproduce experimental phase relations and amplitude ratios. In a subsequent step we closed the loop fine-tuned it to adjust the amplitudes. The conversion to the nonlinear system was done systematically, by comparing the behavior of a cosine function driving the system, to that of the Hill equations, linearized around the steady state (Taylor expansion of the Hill equations). Finally the system needs to be fine-tuned which requires minor optimization of the parameters. In summary we have a parameter search pipeline which is based on a LTI method but adapted to our needs. Below we describe further details how variables and parameters were chosen.

**Choice of Hill functions (Text S1)**

In constructing our model, we made considerable effort to describe the biological processes based on current experimental knowledge to make the model readable to experimentalists and to provide parameters/variables which can be measured. However, many processes are not yet fully understood in quantitative detail. For example, while it is well accepted that PER/CRY inhibits CLOCK/BMAL mediated transcription, it is not yet clear how this can be achieved on a molecular and quantitative level. In our model we have assumed that PER/CRY acts via CLOCK/BMAL and therefore its inhibitory action can only be carried out in the presence of CLOCK/BMAL. This leads to the term in the Hill function “*(PC/ki3)(x1/kt3)*”- see Text S1, equation 2 - where *PC* represents the pool of PER/CRY complexes and *x1* refers to CLOCK/BMAL. This sort of term is a non-trivial type of the Hill equation – nevertheless, in our opinion it describes more accurately our biological knowledge of the system. Such a kinetics describing the action of the inhibitor in the presence of an activator can be derived directly from biophysical laws [3]**.** As a consequence, such Hill-type equations are used to describe transcription reactions including complicated combinatorial regulation as well as epigenetic regulations.

**Choice of different Hill coefficients**

The variables in the model represent the combined action of several paralogs (e.g. *Per* represents *Per1*, 2, 3) and each promoter may contain different cis-regulatory elements [4,5,6,7]. For these reasons, we adapted the Hill coefficients (Table 2C, Text S1) to the effect which is observed in the expression pattern of the particular genes (amplitude and phase of the RNA for the given gene). This is biologically not unreasonable, since the activation kinetics of different genes might well vary due to several reasons: i) there may be different number of E-boxes in the promoter regions of the genes in the model (defining active E-boxes is experimentally demanding and the reported numbers vary within the literature [4,8,9]), ii) the Hill coefficients used are *effective values* and also describe cooperativity effects and chromatin modifications such as histone acetylation and methylation. The fact that different Hill coefficients reproduce the data might reflect potentially different actions of CLOCK/BMAL on the different genes.

**Posttranslational modifications (Text S1, Table1)**

As for the genes also the proteins and protein complexes considered in the model represent the combined action of several paralogs. Our choice of explicitly representing certain complexes or certain phosphorylated states is related to the biological situations we were interested in. In principle posttranslational modifications of other proteins could be included in extended model versions such as modifications of CLOCK [10], BMAL [11], REV-ERB [12]. Such posttranslational modifications are of general interest and can be incorporated in the model at a later stage.

Other variables are not represented as differential equations but play a role in the system implicitly. These are CLOCK and PC. 1) CLOCK is used in the model as an auxiliary protein which helps to understand the system from the biological point of view. Therefore, the formation of CLOCK/BMAL complex is only virtual and rather corresponds to an activation of BMAL. 2) PC represents a pool of the two variables *x2* and *x3* (see Text S1 equation (20)). We created it to facilitate the reading/writing of the equations and the figures (Figure 1 and Figure S1). This “variable” is also used when replacing the PC loop by a constitutive inhibitor (Figure 3 B). The effect of different inhibitor (PC) strengths is depicted in Supplementary Figure 2 (this figure represents an extension of the studies presented in Figure 3 B). In the Figure we show 6 *in silico* experiments were we perturb the PC wild type mean value (*PC_WT_*=1.7) to +/- 10%, +/- 20%, +/- 50%. As shown in the figure the oscillations are not lost. The results of these simulations also strengthen our concept of an independently oscillating RBR loop.

**Degradation rates for mRNAs and proteins (Text S1, Table 2A)**

The degradation rates in the system (*d*) were determined using the linear model as described above. We took as starting point the experimental phase difference between variables (Dataset S1 Table 2) and determined the degradation parameters accordingly (see example at the end of this supplement). The values found were fine-tuned as described to allow the maintenance of similar phase differences in the nonlinear version of the model. Our calculated values for degradation rates (*Per*, *Cry* and *Ror*) are consistent with independently published half-lives (see Dataset S1, Table 3). However, published measurements of rate constants sometimes vary considerably depending on the cell type, tissue and even experimental method used (e. g. published values for *Per2* degradation rate are between 0.87 and 3.04 - Dataset S1 Table 3). In addition, we consider our parameters as *effective values* which often represent a set of genes/proteins with different degradation rates or take into account several factors. As an example, the degradation rate of *Rev–Erb* represents a basal degradation together with a self-inhibition [13] and is therefore set higher in our model than experimentally reported. In the case of *Bmal1* mRNA half-life, a recent publication [14] (reporting values between 40 and 100 minutes) supports our choice for the model (Dataset S1 Table 3).

**Reaction rates for complex formation/dissociation (Text S1, Table 2A)**

The rates regarding the complex CLOCK/BMAL, which represents an activated form of BMAL, were chosen to fit the overall amplitudes of the closed linear loop in comparison to the open linear loop. Moreover we considered that the amount of active BMAL is much higher than the non-active form. For the all formation/dissociation rates of the different PER/CRY complexes we assumed equal values.

**Reaction rates for phosphorylation/dephosphorylation (Text S1, Table 2A)**

The value for the phosphorylation rate *kph_z2_* was taken according to Vanselow *et al.* [15]. We considered the rate of dephosphorylation to be much smaller implying that most PER will be phosphorylated.

**Transcription rates (Text S1, Table 2A)**

Values for the transcription rates are calculated in the non-linear version of the model using a Taylor expansion of the nonlinear transcription equation around the steady state and comparing the equations with its linearized version.

**Activation/inhibition and production rates (Text S1, Table 2B)**

These rates could be calculated as described above (Degradation rates for mRNAs and proteins (Table 2A Text S1) and fine-tuned in the nonlinear model.

**Transcription fold activation (Text S1, Table 2B)**

The fold activation parameter describes the asymptotic increase achieved for the full activation. It can be measured by studying saturated activation. The parameter was inserted based on a publication by Sato *et al* [16], were the fold activation of *Bmal* by ROR is measured. Therefore, we introduced this parameter that could be eventually measured for other clock components.

**Import/export rates (Text S1, Table 2B)**

The import rates for REV-ERB_C_, ROR_C_ and BMAL_C_ were calculated following the same pipeline described above. Regarding the PER/CRY complexes some assumption were made based on knowledge from our own experimental data [15,17]: 1) The PER*/CRY complex enters the nucleus faster than its unphosphorylated form; 2) there is no accumulation of the proteins in the cytoplasm and therefore the export rate of all complexes to the cytoplasm is much smaller than the corresponding import rate to the nucleus.

**Exogenous RNA (Text S1, Table 2C)**

These parameters (*y1_0_*, *y2_0_*, *y3_0_*, *y4_0_*, *y5_0_*) are set to zero and therefore play no role in the wild type system. However, it is important to simulate overexpression experiments were constitutive exogenous RNA is added to the system (Figure 7).

As an example we show the calculations for the following parameters (see Text S1 for identification of the parameters): *k_p3_*, *ki_z6_*, *d_x5_* and *d_z6_*. The production and import of REV-ERB can be described as in reaction (1):

(11)

were *CT* is the circadian time for the indicated gene or protein and *Anorm* is the normalized amplitude (see Dataset S1 Table 2).

We assume that *d1* is higher than *d2* (*d1*=5*d2*) and via equations (8) and (9) we obtain,

$\arctan\left(\frac{w}{5\cdot d2}\right)+\arctan\left(\frac{w}{d2}\right)=(9-4)\cdot\frac{2\pi}{24}$ (12)

This leads to *d1* = 0.83 and *d2* = 0.17 and from previous considerations we have *d3* = 0.45. Using equations (5) and (10) we obtain,

$b1\cdot\frac{1}{\sqrt{d1^{2}+w^{2}}}\cdot b2\cdot\frac{1}{\sqrt{d2^{2}+w^{2}}}\cdot b3\cdot\frac{1}{\sqrt{d3^{2}+w^{2}}}=\frac{0.26}{0.54}$ (13)

If we assume *b1* = *b3* = *b* and *b2* < *d1* (*b2* = 0.5) we obtain for *b* the value 0.37.

We could determine the desired parameters by replacing the values calculated with (13) in the equations for REV-ERB_C_ and REV-ERB_N_ (see Text S1 equations (4) and (6)),

*k_p3_* = *b1* = 0.37; *ki_z6_* = b2 = 0.5; *d_x5_* = *d2* = 0.17 and *d_z6_* = *d1* – *b2* = 0.33.

Comparing these values with the ones given in Text S1 tables 2A and 2B we can see that only *dz6* needed to be fine-tuned for better fitting of the full nonlinear model. Its value was changed from 0.33 to 0.31.

This calculation exemplifies how experimental information (phases, amplitudes), reasonable assumptions and linearization allow systematic parameter determination.

**References**

1. Mackey MC, Glass L (1977) Oscillation and chaos in physiological control systems. Science 197: 287-289.

2. Ang J, Ingalls B, McMillen D (2011) Probing the input-output behaviour of biochemical and

genetic systems: system identification methods from control theory. METHODS IN ENZYMOLOGY 487: 279-317.

3. Cha S (1968) A simple method for derivation of rate equations for enzyme-catalyzed reactions under the rapid equilibrium assumption or combined assumptions of equilibrium and steady state. J Biol Chem 243: 820-825.

4. Ueda HR, Hayashi S, Chen W, Sano M, Machida M, et al. (2005) System-level identification of transcriptional circuits underlying mammalian circadian clocks. Nat Genet 37: 187-192.

5. Hida A, Koike N, Hirose M, Hattori M, Sakaki Y, et al. (2000) The human and mouse Period1 genes: five well-conserved E-boxes additively contribute to the enhancement of mPer1 transcription. Genomics 65: 224-233.

6. Travnickova-Bendova Z, Cermakian N, Reppert SM, Sassone-Corsi P (2002) Bimodal regulation of mPeriod promoters by CREB-dependent signaling and CLOCK/BMAL1 activity. Proc Natl Acad Sci U S A 99: 7728-7733.

7. Yamajuku D, Shibata Y, Kitazawa M, Katakura T, Urata H, et al. (2010) Identification of functional clock-controlled elements involved in differential timing of Per1 and Per2 transcription. Nucleic Acids Res 38: 7964-7973.

8. Hamilton EE, Kay SA (2008) SnapShot: circadian clock proteins. Cell 135: 368-368 e361.

9. Guillaumond F, Dardente H, Giguere V, Cermakian N (2005) Differential control of Bmal1 circadian transcription by REV-ERB and ROR nuclear receptors. J Biol Rhythms 20: 391-403.

10. Yoshitane H, Takao T, Satomi Y, Du NH, Okano T, et al. (2009) Roles of CLOCK phosphorylation in suppression of E-box-dependent transcription. Mol Cell Biol 29: 3675-3686.

11. Sahar S, Zocchi L, Kinoshita C, Borrelli E, Sassone-Corsi P (2010) Regulation of BMAL1 protein stability and circadian function by GSK3beta-mediated phosphorylation. PLoS One 5: e8561.

12. Yin L, Wang J, Klein PS, Lazar MA (2006) Nuclear receptor Rev-erbalpha is a critical lithium-sensitive component of the circadian clock. Science 311: 1002-1005.

13. Adelmant G, Begue A, Stehelin D, Laudet V (1996) A functional Rev-erb alpha responsive element located in the human Rev-erb alpha promoter mediates a repressing activity. Proc Natl Acad Sci U S A 93: 3553-3558.

14. Suter DM, Molina N, Gatfield D, Schneider K, Schibler U, et al. (2011) Mammalian genes are transcribed with widely different bursting kinetics. Science 332: 472-474.

15. Vanselow K, Vanselow JT, Westermark PO, Reischl S, Maier B, et al. (2006) Differential effects of PER2 phosphorylation: molecular basis for the human familial advanced sleep phase syndrome (FASPS). Genes Dev 20: 2660-2672.

16. Sato TK, Panda S, Miraglia LJ, Reyes TM, Rudic RD, et al. (2004) A functional genomics strategy reveals Rora as a component of the mammalian circadian clock. Neuron 43: 527-537.

17. Vanselow K (2006) Funktionelle Bedeutung von PERIOD2-Proteindomaenen fuer die Dynamic circadianer Oszillationen. PhD Thesis.
